# Intensive cognitive behavioral therapy for obsessive-compulsive disorder: a meta-analysis of randomized controlled trials

**DOI:** 10.3389/fpsyt.2026.1937285

**Published:** 2026-09-28

**Authors:** Jianli Zhu, Chunxiao Zhang, Xinpeng Xu, Dongsheng Lu, Weigang Pan, Xiaohong Li

**Affiliations:** 1 Department of Psychology, Yulin Veterans’ Hospital, Yulin, Guangxi, China; 2 Department of Psychiatry, Chengdu Zongnan Hospital, Chengdu, Sichuan, China; 3 Peking University Huilongguan Clinical Medical School, Huilongguan Hospital, Capital Medical University, Beijing, China; 4 Department of Psychiatry VII, Changping Hospital of Integrated Traditional Chinese and Western Medicine Hospital, Beijing, China

**Keywords:** cognitive behavioral therapy, intensive treatment, meta-analysis, obsessive-compulsive disorder, randomized controlled trial

## Abstract

**Background:**

Intensive cognitive behavioral therapy (CBT) delivers more or longer sessions over a shorter time than standard CBT. This review aimed to examine the effects of intensive CBT in comparison to non-active conditions and other active treatments for obsessive-compulsive disorder (OCD).

**Method:**

We analyzed standardized mean difference (SMD) at post-treatment and follow-up for OCD symptoms, depression, anxiety, and family accommodation (FA). Moderator analyses and meta-regression were conducted to investigate factors associated with OCD symptom reduction.

**Results:**

Fourteen randomized controlled trials (*n* = 786) were included. At post-treatment, the effect of intensive CBT varied by comparator types. It was significant against non-active/minimal intervention controls (SMD = -1.74, 95% CI [-2.53, -0.95], *p* < 0.001) and not significant against active psychological treatments or pharmacotherapy. Significant improvements were also found for depression, anxiety, and FA. Exploratory meta-regression suggested that higher weekly treatment intensity (hours per week) was associated with greater symptom reduction, although this finding should be interpreted cautiously due to the limited number of studies. Follow-up analyses suggested sustained OCD improvement, while secondary outcome data were limited. The certainty of evidence was low to very low across outcomes at post-treatment.

**Discussion:**

Intensive CBT appears superior to non-active controls, while its advantage over active treatments remains uncertain and the certainty of the evidence is low to very low. The substantial heterogeneity and potential publication bias require cautious interpretation. Future high-quality RCTs with longer follow-up are needed.

**Systematic review registration:**

https://www.crd.york.ac.uk/PROSPERO/, identifier CRD42022365015.

## 1 Introduction

Obsessive-compulsive disorder (OCD) is a prevalent psychiatric disorder characterized by persistent intrusive thoughts and/or ritualized repetitive behaviors (1). It typically emerges in childhood and gradually transitions to a chronic course, leading to significant impairment in social function and poor quality of life (2, 3). A cross-sectional epidemiological survey in China reported a weighted lifetime prevalence of 2.4% and a weighted 12-month prevalence of 1.6% for OCD, ranking it second only to specific phobia among anxiety disorders (4). Providing effective and timely treatment for patients with OCD is therefore essential.

Cognitive behavioral therapy (CBT), with exposure being a fundamental component, is the current first-line evidence-based psychological treatment for anxiety and obsessive-compulsive disorders. Standard CBT usually consists of 10 to 18 weekly one-hour sessions, employing a step-by-step approach tailored to the specific disorder (5). Previous meta-analyses have indicated that standard CBT leads to a reduction in symptoms with a moderate effect size, response rates of approximately 70%, and remission rates around 50% for OCD (5, 6). There is evidence supporting that CBT has significantly larger effect sizes than placebo, waiting list and pharmacotherapy for patients with OCD (7, 8). However, approximately 50% of patients with OCD who undergo standard CBT fail to fully respond (1). Consideration of stepped care models such as that described in the National Institute of Clinical Excellence guidelines (2005) provides recommendations for more intensive treatment for treating non-responders.

Intensive CBT shares many similarities with standard CBT, but it is characterized by a significantly higher frequency of sessions (9). While standard CBT typically spans 12 or more weeks, intensive CBT involves a shorter total treatment duration of less than six weeks and a more frequent session schedule. Research indicates that intensive CBT demonstrates comparable effectiveness to standard CBT while requiring only half the time commitment (10, 11). Intensive CBT has primarily been applied to anxiety disorders and has demonstrated effectiveness in treating generalized anxiety disorder, panic disorder, and social anxiety disorder. However, limited literature addresses its efficacy for OCD (12).

Jónsson et al. (13) conducted a meta-analysis comparing intensive CBT with standard CBT for youth and adults with OCD, revealing that intensive CBT yielded larger effects immediately post-treatment, although this advantage did not persist at follow-up. This finding aligns with evidence suggesting that intensive treatments provide more immediate relief than standard treatments for adults experiencing severe and acute anxiety, accompanied by lower dropout rates (14). Other meta-analyses have indicated that both intensive and standard CBT are comparable in their effectiveness for youth and adults with anxiety disorders and OCD at post-treatment, with these results being maintained during follow-ups (12, 15). Moreover, the Bergen 4-day Treatment (B4DT) has shown high recovery rates at one- to four-year follow-ups, providing reliable evidence of the long-term efficacy of intensive CBT for patients with OCD (16–18). In line with the trends towards reducing treatment duration and increasing session frequency, intensive CBT offers advantages that rival those of standard CBT, particularly for patients with persistent OCD (19–21). The intensive format appears to be both effective and acceptable for patients with OCD. Notably, all youth-focused meta-analyses prioritized the examination of clinical symptoms and remission rates while systematically excluding studies involving alternative therapeutic approaches, collective treatment formats, and non-randomized designs (12, 13, 22).

To the best of our knowledge, this is the first meta-analysis specifically focused on intensive CBT for OCD, based exclusively on RCTs. RCTs were eligible for inclusion, addressing the issue of lack of control conditions in existing meta-analyses and increasing validity (13). Previous systematic reviews have predominantly concentrated on standard CBT, with only a few studies examining the novel approach of intensive treatment delivery (23–25). Öst and Ollendick (12) investigated the acceptance and effects of intensive CBT for youth with anxiety disorders, but only three studies on OCD were included, providing limited support for the effectiveness of intensive CBT in reducing OCD symptoms. Meta-analysis involving youth with anxiety disorders or OCD found no significant post-treatment differences in symptom severity between intensive CBT and standard treatment (26). However, challenges remain in assessing the efficacy of intensive CBT among adult patients. Jónsson (13) conducted a meta-analysis to assess the efficacy of intensive CBT for youth and adults with OCD, utilizing pre-post comparisons to evaluate effect sizes. However, correlations between baseline and post-test symptom scores could potentially bias the outcomes (27). While it can be concluded that intensive CBT is effective for OCD to some extent, its comparative effectiveness with other treatments remains unclear. Therefore, a meta-analysis based on randomized controlled trials (RCTs) is crucial for providing information on the effectiveness of intensive CBT.

## 2 Materials and methods

The purpose of this study was to examine the efficacy of intensive CBT for patients with OCD, using data extracted from RCTs at post-treatment and follow-up. Primary outcomes included OCD symptom scores assessed with validated assessment tools. Subgroup analyses by age (children/adolescents vs. adults) was conducted to determine whether the treatment effect differed between age groups. Additionally, we conducted exploratory analyses to investigate whether gender, comorbidity, medication use, study quality, and variables related to treatment intensity would moderate the effect sizes of OCD symptom improvements. Secondary outcomes included anxiety, depression, and family accommodation (FA) scores, which are widely considered important indicators for OCD treatment success (9–11).

### 2.1 Protocol and registration

This meta-analysis was pre-registered with the International Prospective Register of Systematic Reviews (PROSPERO: registration number CRD42022365015) as a meta-analysis of intensive cognitive behavioral therapy for OCD.

### 2.2 Research strategies

We systematically searched both English (Cochrane, Embase, Medline, PubMed, PsycINFO, and Web of Science) and Chinese databases (CNKI and WanFang), on May 6 and May 13, 2026. We screened systematic reviews and meta-analyses on intensive CBT for OCD to identify additional relevant studies. The search strategy is (obsess* OR compul*) AND ((cognitive behavior* AND (therap* OR psychotherap* OR treatment OR intervention)) OR exposure response prevention) AND (intensive* or concentrate* or short* or short-term or week* or day or daily or quick*) AND (random* OR control* OR placebo* OR blind*). More details are shown in **Supplementary Table 1**.

### 2.3 Eligibility criteria

Two reviewers [CX and JL] screened initial eligible articles based on titles and abstracts. A third reviewer was consulted to resolve any discrepancies, and CX and JL subsequently assessed the full texts of potentially eligible studies. Articles were included if they met the following criteria: (a) were original, peer-reviewed RCTs; (b) were written in English or Chinese; (c) included children, adolescents, or adults with a primary OCD diagnosis according to standardized diagnostic criteria (ICD or DSM); (d) evaluated the efficacy of an intensive CBT intervention; (e) used validated OCD symptom measures; (f) reported sufficient quantitative data to calculate effect sizes. We excluded non-randomized studies, secondary analyses, remote-delivered therapies (e.g., internet-based, videoconferencing, or telephone-based CBT), studies not specifically designed to evaluate the efficacy of intensive CBT (e.g., neuroimaging studies without clinical outcome comparison), and articles without original data (e.g., reviews, case reports, editorials, and commentaries).

In this study, we distinguished between the definition of intensive CBT (i.e., what constitutes “intensive”) and additional eligibility criteria applied during study selection to ensure clinical homogeneity. Intensive CBT was operationally defined as a minimum of 12 hours of CBT delivered within a maximum total treatment duration of six weeks (12, 13, 28). Additional eligibility criteria: included studies were also required to use ERP as the primary therapeutic technique, while studies primarily based on third-wave approaches such as acceptance and commitment therapy were excluded (29). Delivery format (individual, group, or family-based) was not part of the intensity definition but was considered during study selection to reflect the range of formats commonly used in clinical practice. Instead of excluding intensive CBT with or without concomitant pharmacotherapy, we accounted for medication use as a moderator.

### 2.4 Data extraction and outcomes

Reviewers XP and DS independently reviewed and extracted data from all eligible full-text articles. Any disagreements were resolved by a third reviewer (XHL). Study descriptions included authors’ names, publication years, countries, diagnosis criteria, comorbidity, age, gender, and other relevant details. The primary outcome measures were OCD symptoms at baseline and end of treatment, assessed with the Yale-Brown Obsessive Compulsive Scale (Y-BOCS), Children Yale-Brown Obsessive Compulsive Scale (CY-BOCS) (30, 31). Our secondary outcomes were symptom severity of anxiety, depression, and FA measured on any validated scale.

### 2.5 Risk of bias evaluation

Two reviewers (CX and JL) independently assessed the risk of bias using the Cochrane Handbook for Systematic Reviews of Interventions across six dimensions: random sequence generation and allocation concealment (selection bias), blinding of participants and personnel (performance bias), blinding of outcome assessment (detection bias), incomplete outcome data (attrition bias), and selective reporting (reporting bias) (32). Trials were rated as high, low, or unclear risk of bias according to the guideline. Performance bias and detection bias were assessed independently, and detection bias was rated based on blinding of outcome assessors. The Jadad scale was used to assess study quality, with scores ranging from 0 to 5 based on randomization, blinding, and drop-out reporting (33). Disagreements were resolved through discussion or consulting a third reviewer (XHL).

The grading of recommendations assessment, development and evaluation (GRADE) system was used to judge the evidence level of primary outcomes (34, 35). We followed the downgrading guidance updated on GRADEpro (https://www.gradepro.org/). The following criteria were applied for downgrading decisions across all outcomes: (a) Publication bias: downgraded when funnel plot asymmetry was observed. (b) Imprecision: downgraded when the total sample size fell below the optimal information size (OIS: <400 participants for continuous outcomes) or when the confidence interval crossed the decision threshold. (c) Risk of bias: downgraded once when most information (more than two-thirds of the weight) came from studies with unclear risk of bias; downgraded twice when most information (more than two-thirds of the weight) came from studies with high risk of bias. (d) Inconsistency: downgraded based on the I^2^ statistic; I^2^ between 50% and 75% led to a one-level downgrade, and I^2^ ≥ 75% led to a two-level downgrade. (e) Indirectness: considered when there were substantial differences in population, intervention, or outcome measures; this was not a major source of downgrading.

### 2.6 Data synthesis and meta-analysis

We used Cochrane’s Review Manager Version 5.4 (http://www.cochrane.org) and Comprehensive Meta-Analysis V3 (https://www.meta-analysis.com) to analyze data. For continuous outcomes measured by different scales, we calculated the standardized mean difference (SMD) as the effect size, computed by dividing the mean difference (MD) in post-treatment scores by the pooled standard deviation (36, 37). All SMDs were reported as Hedges’ g to correct for small-sample bias, as implemented in RevMan. A negative SMD indicated a favorable effect for intensive CBT relative to control groups. A random-effects model with the DerSimonian-Laird estimator was selected *a priori* due to anticipated clinical and methodological heterogeneity among included studies (38). Heterogeneity was quantified using the *I^2^* values > 50% were considered indicative of substantial heterogeneity (39–41). For multi-arm studies, we followed the approach recommended in the Cochrane Handbook. The sample size of every arm reused across comparisons was divided appropriately, while the group mean and standard deviation remained unchanged. For example, when a shared intervention group was compared against multiple control groups, its sample size was divided across the comparisons; when a control group was shared across multiple comparisons, the same principle was applied. No imputation was performed for missing data; analyses at each time point included only studies that reported the required data at that specific time point. When multiple follow-up assessments were reported, we extracted the longest available follow-up for the primary analyses.

Subgroup analyses were done to seek the effects of three control types: non-active condition (e.g., waiting-list or psychological placebo), psychological treatment (e.g., standard CBT), and pharmacotherapy (e.g., SRIs, SNRIs). TAU was considered a non‑active/minimal intervention control if it consisted of non‑standardized general clinical management, supportive therapy, or no treatment, without systematic ERP or CBT. Conversely, TAU was classified as an active psychological treatment if it explicitly included manualized ERP or CBT as a systematic intervention. In addition, subgroups analyses by age group were performed to examine the moderating effect of age on treatment outcomes.

### 2.7 Exploratory analyses

Given that subgroup analyses may overlook potential interactions among moderators, we pre-specified a series of exploratory analyses to examine factors that could moderate treatment effects. We coded the following dichotomous variables: (a) female predominance (> 50% vs. ≤ 50%), (b) proportion of patients with comorbidity (> 50% vs. ≤ 50%), (c) Jadad score (> 3 vs. ≤ 3), and (d) medication use during intensive CBT (yes vs. no).

Meta-regression analyses were planned to examine the impacts of treatment time and intensity: (a) session duration, (b) total sessions, (c) total treatment hours, (d) total treatment weeks, and (e) weekly treatment intensity (hours per week). Given the high inter-correlation among these variables, we planned to enter them into separate univariable regression models rather than a single multivariable model. Appropriate numbers of studies for meta-regression are more than ten (40, 41). We considered a *p* of ≤ 0.05 for other outcomes as a threshold for statistical significance, and all meta-analytic outcomes were two-tailed (42). All meta‑regression and moderator analyses were pre‑specified as exploratory. No adjustment for multiple testing was applied, as the analyses were designed to generate hypotheses rather than to test confirmation.

### 2.8 Sensitivity analyses

To detect the consistency of the effects of intensive CBT for patients with OCD, we conducted a leave-one-out sensitivity analysis, sequentially removing each study (43). Using CMA, we excluded one study at a time to determine if the effect sizes remained consistent (44). To further assess the robustness of our findings to the handling of multi-arm trials, we conducted an additional sensitivity analysis in which we included independent comparisons per multi-arm study. In addition, we performed a sensitivity analysis based on study quality. We performed a sensitivity analysis by excluding studies with low methodological quality (two or more domains of risk of bias are rated as high risk). The pooled effect size was recalculated after removing these studies.

### 2.9 Publication bias

Publication bias was assessed using visual inspection of funnel plots and Egger’s regression test, with results interpreted cautiously given the recommended minimum of 10 studies for reliable funnel plot asymmetry testing (45–47). When Egger’s regression intercept approaches zero and the *p*-value is not significant, the possibility of publication bias is limited (48). We also calculated the fail-safe N as additional sensitivity information, although this metric cannot determine whether publication bias exists and should not be used to counter the asymmetry findings from funnel plots or Egger’s test (49). For multi-arm studies, we included only one comparison per study, typically intensive CBT versus the non-active control condition, to avoid violating the independence assumption underlying funnel plot interpretation. All publication bias analyses were treated as exploratory and interpreted with caution.

## 3 Results

### 3.1 Study selection and characteristics

A total of 6363 relevant articles were identified, consisting of 5811 English articles and 552 Chinese articles. We performed the meta-analysis in accordance with the Preferred Reporting Items for Systematic Reviews and Meta-Analyses (PRISMA) (50). The screening process is illustrated in the flow diagram (**Figure 1**). After full-text screening, fourteen studies remained. Two RCTs lacked post-treatment data but reported follow-up assessments; these were included only in the follow-up analyses. One study was excluded because it was not a RCT, despite being initially considered as such (10, 51–64) (**Supplementary Table 1**). In Challacombe et al. (54) and Lewin et al. (57), TAU consisted of non‑specific counseling, mindfulness, medication, or no treatment; these were therefore classified as non‑active/minimal intervention controls. Medication use during intensive CBT varied across studies and was examined as a moderator in exploratory analyses. Medication changes during follow-up were not systematically reported.

**Figure 1:**
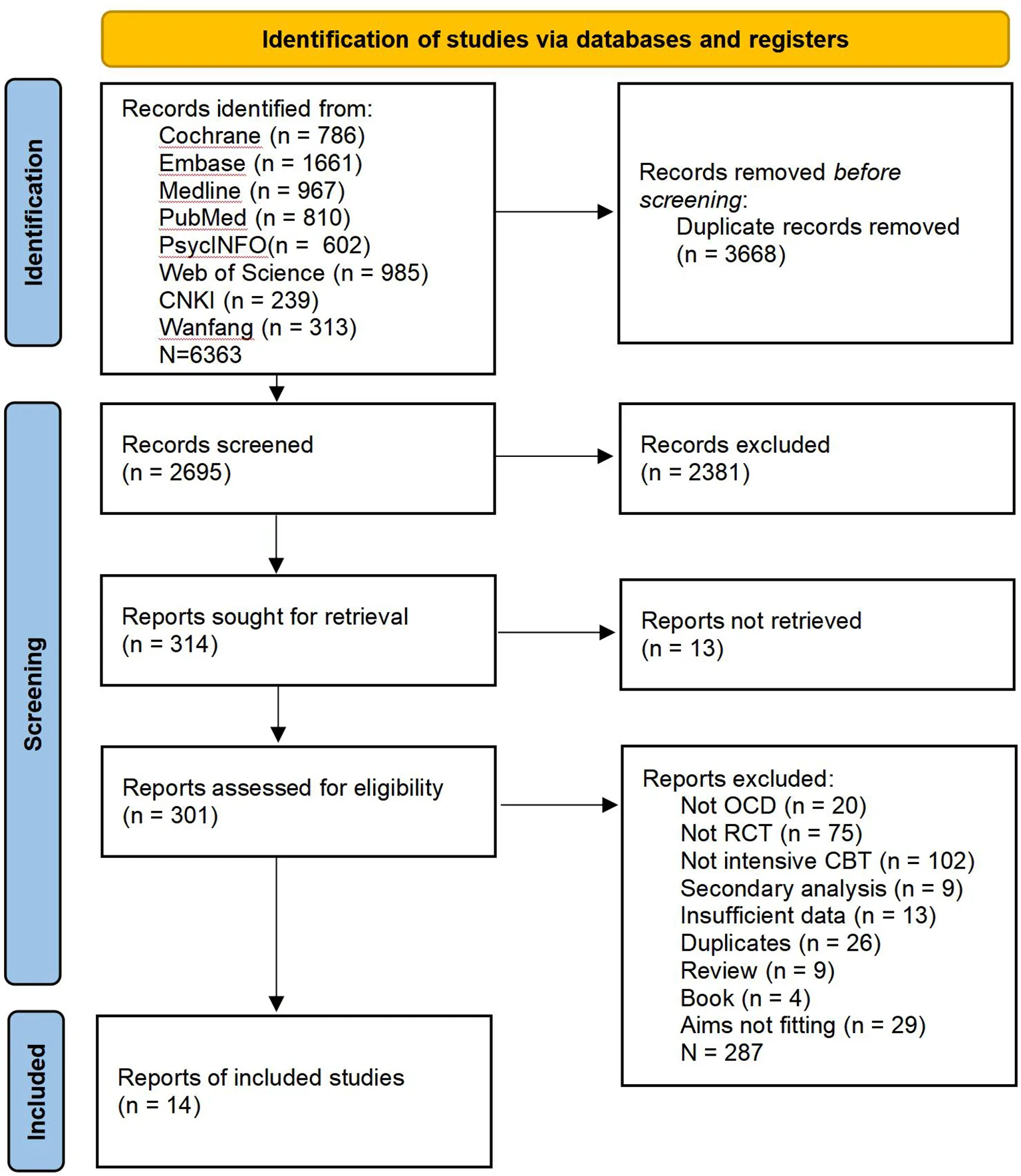
PRISMA flow diagram.

### 3.2 Risk of bias and quality assessment

**Figure 2** presents the risk-of-bias summary of the fourteen studies. One study was rated as high risk of selection bias due to lack of information about random sequence generation and allocation concealment. One study was rated as high risk of detection bias, one for attrition bias, and one for reporting bias. All studies were rated as high risk of performance bias, as blinding of participants and therapists is not feasible in psychotherapy trials. The Jadad scores ranged from 2 to 5. Detailed trial characteristics and quality assessments are presented in **Supplementary Table 2**.

**Figure 2:**
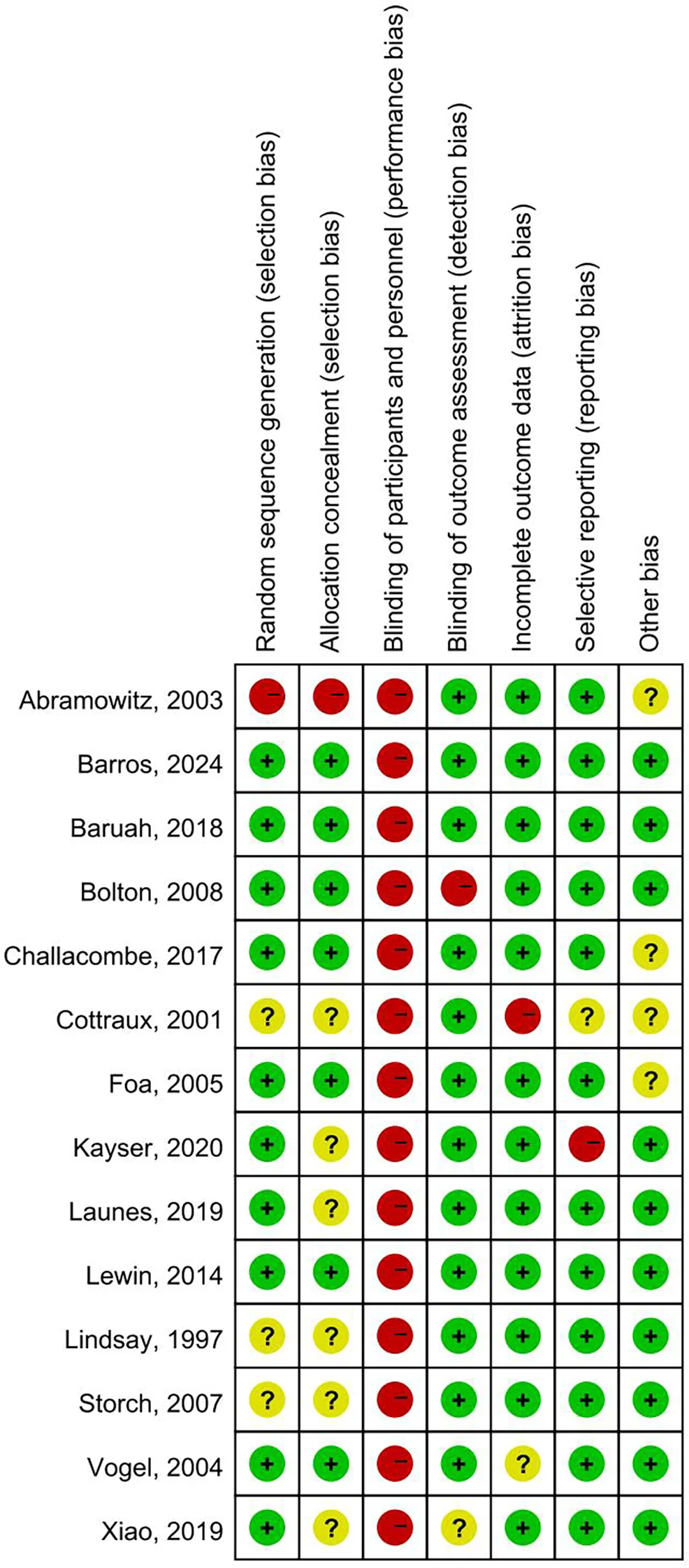
Risk of bias.

### 3.3 Primary outcome

#### 3.3.1 Post-treatment OCD symptoms

Twelve RCTs provided post-treatment Y-BOCS/CY-BOCS data. In the forest plot, *Launes 2019a* denotes the comparison of intensive CBT versus waiting list, and *Launes 2019b* denotes the comparison of intensive CBT versus self-help. Because different control conditions represent fundamentally different clinical contrasts, the overall pooled effect size (SMD = -1.00, 95% CI [-1.43, -0.56], *p* < 0.001) should be interpreted with caution. The main findings were derived from subgroup analyses stratified by comparator type (**Figure 3**). Non-active/minimal intervention controls (waiting list, self-help, psychological placebo; k = 7) showed a significant effect favoring intensive CBT (SMD = -1.74, 95% CI [-2.53, -0.95], *p* < 0.001, *I*^2^ = 81%). Active psychological treatment controls (standard/weekly CBT, cognitive therapy; k = 4) yielded a small, non-significant effect (SMD = -0.17, 95% CI [-0.47, 0.14], *p* = 0.28, *I*^2^ = 5%). There was no evidence of a difference between pharmacotherapy controls (SSRIs, SNRIs e.g., k = 2) and intensive CBT (SMD = -0.72, 95% CI [-1.61, 0.16], *p* = 0.11, *I*^2^ = 49%).

**Figure 3:**
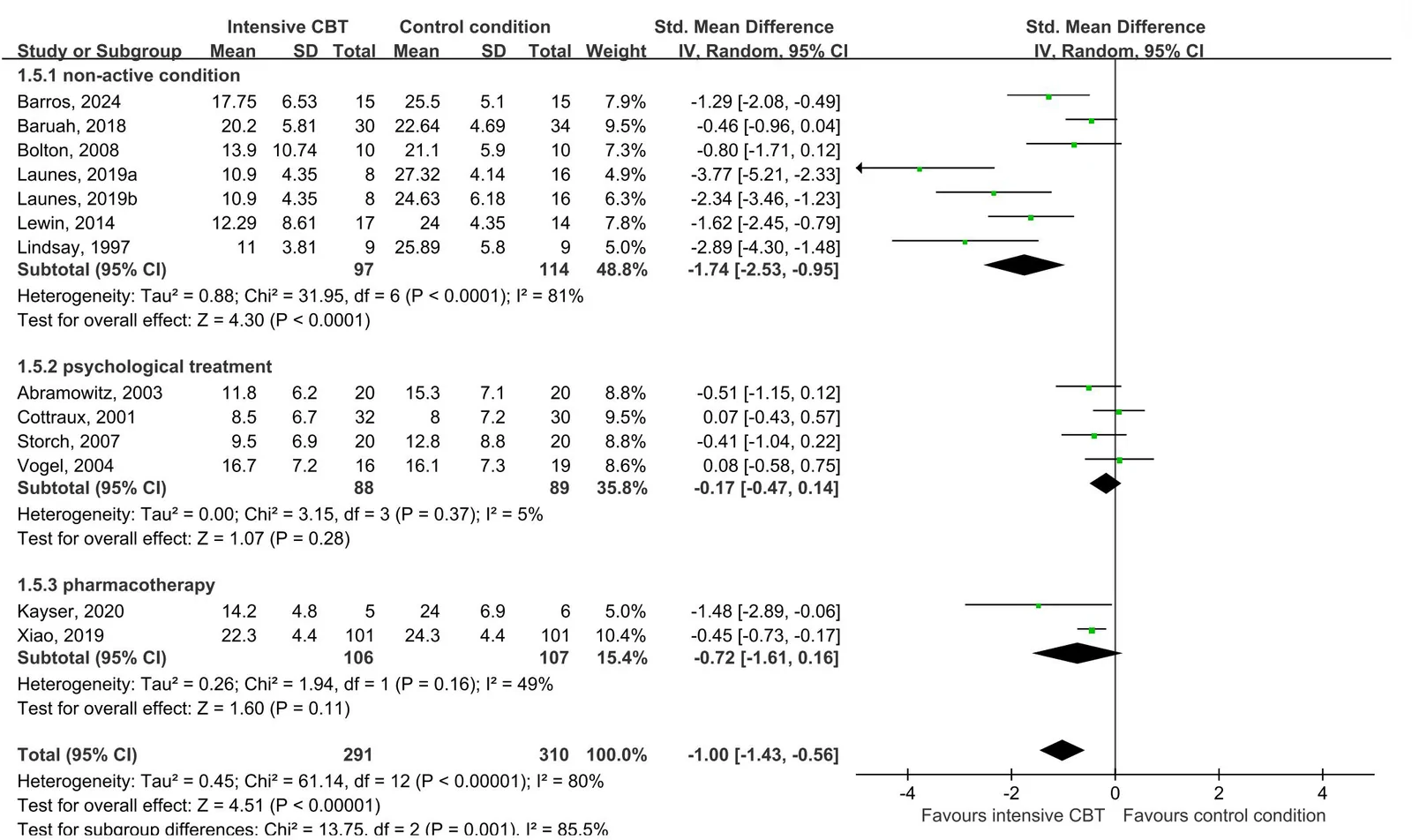
Effects of intensive CBT vs. control interventions on OCD symptoms at post-treatment.

The *I^2^* was 80% (*χ^2^* = 61.14, *p <* 0.001), suggesting substantial heterogeneity. A leave-one-out sensitivity analysis yielded effect sizes ranging from -1.18 to -0.87 (*p* < 0.001), confirming that no single study unduly influenced the pooled estimate. Excluding studies with high risk of bias (two or more domains of risk of bias are rated as high risk) resulted in a similar effect (SMD = -1.31, 95% CI [-1.92, -0.70], *p* < 0.001, k = 9), indicating that the findings were not driven by a single study quality concern (51, 57). A sensitivity analysis including only one independent comparison in multi-arm study revealed a similar result (**Supplementary Figures 1**–**3**). The funnel plot showed some asymmetry, and Egger’s regression test suggested publication bias (intercept = -3.52, *p* < 0.01), indicating possible small-study effects, one potential explanation for which is publication bias (**Supplementary Figure 4**). The classic fail-safe N indicated that was 217, but this metric cannot determine whether publication bias exists and should not be interpreted as counterevidence to the asymmetric funnel plot or significant Egger’s test. The results should therefore be interpreted cautiously.

Subgroup analyses by age showed that the effect of intensive CBT was significant in both children (SMD = -1.00, 95% CI [-1.56, -0.43], *p* < 0.001, *I*^2^= 51%) and adults (SMD = -1.03, 95% CI [-1.59, -0.46], *p* < 0.001, *I*^2^= 84%). The test for subgroup differences was non-significant (*χ^2^* = 0.01, *p* = 0.94, *I*^2^= 0%) (**Supplementary Figure 5**).

#### 3.3.2 Follow-up OCD symptoms

We also tested the efficacy of intensive CBT versus different control conditions at follow-up, using the mean and SD of OCD symptoms provided by eight RCTs. In the forest plot, Foa’s trial contributed four separate comparisons: (a) intensive CBT versus placebo; (b) intensive CBT versus clomipramine; (c) intensive CBT plus clomipramine versus placebo; and (d) intensive CBT plus clomipramine versus clomipramine. The intensive CBT group sample size was split because it was shared between the placebo and clomipramine comparisons; the placebo group sample size was also split because it was shared between the intensive CBT and intensive CBT + clomipramine comparisons; the same principle was applied to other groups. Results indicated that intensive CBT performed better in terms of long-term effects than non-active conditions (SMD = -0.97, 95% CI [-1.35, -0.59], *p* < 0.001) and pharmacotherapy (SMD = -1.01, 95% CI [-1.56, -0.45], *p* < 0.001). There was no significant group difference between intensive CBT and psychological treatments (SMD = 0.08, 95% CI [-0.24, 0.40], *p* = 0.61), and the total SMD synthesized by random-effects model was significant (SMD = -0.59, 95% CI [-0.98, -0.21], *p* < 0.001) (**Figure 4**).

**Figure 4:**
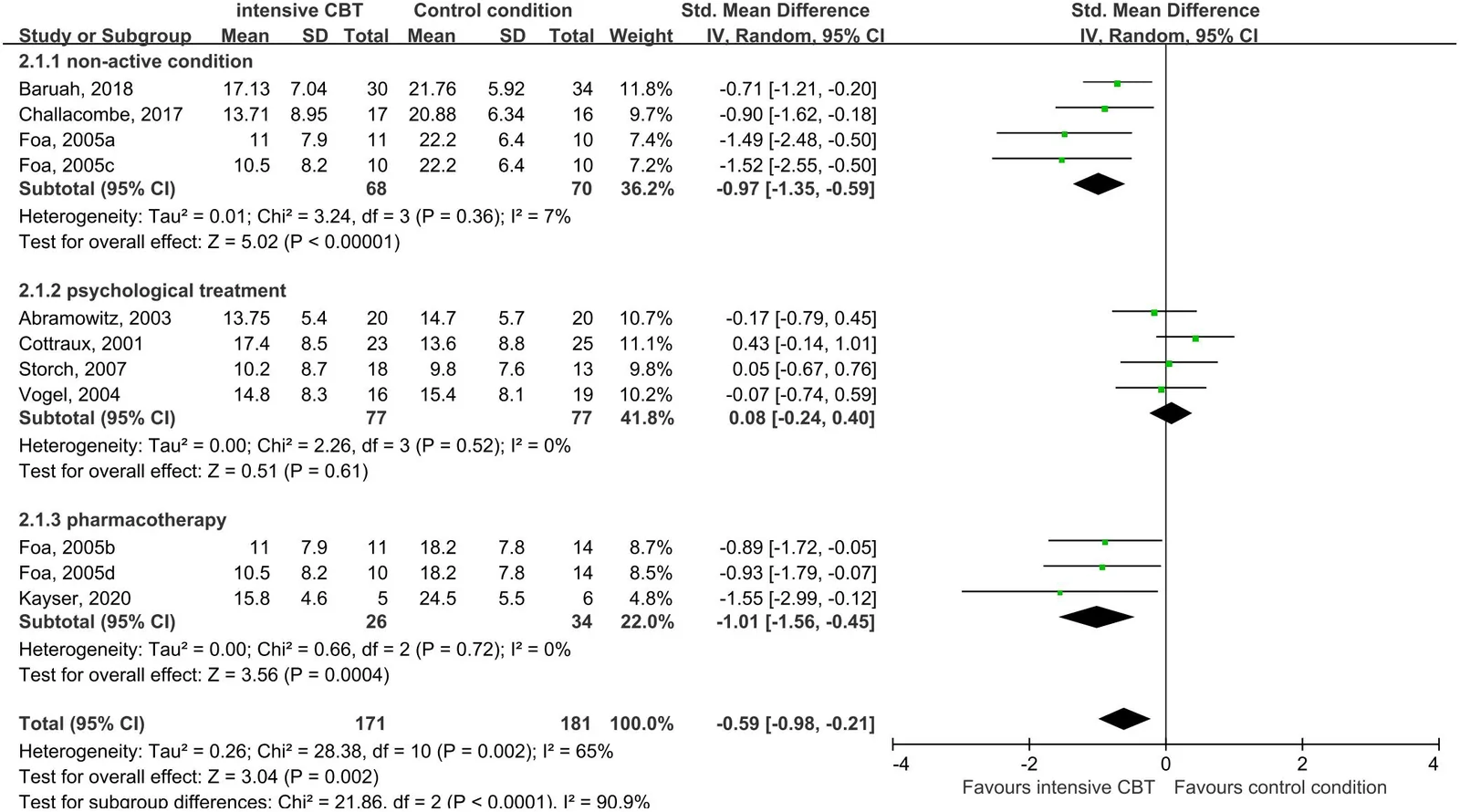
Effects of intensive CBT vs. control interventions on OCD symptoms at follow-up.

The *I^2^* was 64% (*χ^2^* = 28.38, *p <* 0.01), suggesting substantial heterogeneity. Leave-one-out sensitivity analysis yielded consistent effect sizes (range: SMD -0.73 to -0.55, *p* < 0.01), confirming the consistency of the findings. Including independent comparisons per multi-arm study yielded consistent results (**Supplementary Figures 6**–**7**). For follow‑up analyses (k = 8), the funnel plot appeared roughly symmetrical (**Supplementary Figure 8**), and Egger’s regression test was not significant (intercept = -3.18, *p* = 0.36). The classic fail‑safe N was 25. These findings should be interpreted with caution given the small number of follow‑up studies.

Subgroup analyses by age revealed no significant effect in children (SMD = -0.02, 95% CI: [-0.50, 0.47], *p* = 0.95, *I*^2^= 0%), whereas a significant effect was observed in adults (SMD = -0.75, 95% CI [-1.20, -0.30], *p* = 0.001, *I*^2^= 67%). The test for subgroup differences was significant (*χ^2^* = 4.72, *p* < 0.05, *I*^2^= 78.8%), suggesting that age moderated the long-term treatment effect (**Supplementary Figure 9**).

### 3.4 Secondary outcome

#### 3.4.1 Post-treatment anxiety, depression, FA symptoms

For depression, seven studies (*n* = 272) revealed significantly lower levels in the intensity CBT group (SMD = -0.47, 95% CI [-0.87, -0.06], *p* < 0.05). For anxiety, six studies (*n* = 225) also showed a significant reduction after intensive CBT (SMD = -0.59, 95% CI [-1.14, -0.04], *p <* 0.05). For FA scores, four studies (*n* = 165) demonstrated greater efficacy for intensive CBT than control groups at post-treatment (SMD = -0.76, 95% CI [-1.32, -0.21], *p <* 0.01) (**Supplementary Figures 10**–**12**).

#### 3.4.2 Follow-up anxiety, depression, FA symptoms

For follow‑up secondary outcomes, four studies (*n* = 153) showed no significant difference in depression levels between intensive CBT and control groups (SMD = -0.13, 95% CI [-0.45, 0.18], *p* = 0.41). Two studies (*n* = 65) provided data on anxiety symptoms, also showing no significant reduction (SMD = 0.28, 95% CI [-0.21, 0.78], *p =* 0.26). Similarly, two studies (*n* = 95) showed that there was no significant difference in FA scores between groups at follow‑up (SMD = -0.60, 95% CI [-1.54, 0.33], *p* = 0.20) (**Supplementary Figure 13–15**).

### 3.5 Exploratory analyses

#### 3.5.1 Moderated analyses

Subgroup analyses were conducted to explore potential moderators of the treatment effect. No significant subgroup differences were found for female predominance, Jadad score, comorbidity, or medication use. The pooled effect remained significant across all subgroups except for the low-quality subgroup (*p* = 0.06) and non-medication subgroup (*p* = 0.19). Overall, no significant moderators were identified (**Table 1**), suggesting that the beneficial effect of intensive CBT was generally consistent across patient and study characteristics.

**Table 1:** Moderated effects for intensive CBT on OCD symptoms.

Moderators	Heterogeneity			k	95% CI			Test of null (2-Tail)	
	*Q*	*df*	*p*		Point estimate	Lower limit	Upper limit	*Z*	*p*
Female predominance	1.72	1.00	0.19						
> 50%				7	-1.42	-2.12	-0.73	-4.00	0.000
≤ 50%				6	-0.76	-1.46	-0.06	-2.12	0.034
Comorbidity	2.63	1.00	0.11						
> 50%				5	-1.40	-2.10	-0.69	-3.87	0.000
≤ 50%				7	-0.65	-1.21	-0.09	-2.27	0.023
Jadad score	1.99	1.00	0.16						
> 3				8	-1.35	-1.96	-0.75	-4.39	0.000
≤ 3				5	-0.68	-1.39	0.03	-1.88	0.060
Medication	0.99	1.00	0.32						
No				3	-0.65	-1.63	0.32	-1.32	0.188
Yes				10	-1.22	-1.78	-0.67	-4.33	0.000

Q indicated the difference in mean effect size across subgroups, k = number of contrasts informing a particular analysis. *p < 0.05.

#### 3.5.2 Meta-regression

Meta-regression analyses were conducted to examine whether treatment intensity variables moderated the effect of intensive CBT on post-treatment OCD symptoms. Results showed that weekly treatment intensity was significantly associated with effect size (*b* = -0.06, *p* < 0.05, *R*^2^ = 0.43), suggesting an exploratory study-level association between higher weekly treatment intensity and greater symptom reduction. Other intensity variables, including session duration, total sessions, total hours, and total treatment duration (weeks) were not statistically significant. Given the small number of studies and the exploratory nature of these analyses, this association should be interpreted cautiously and does not imply a causal relationship (**Table 2**).

**Table 2:** Continuous variables as potential moderators of OCD symptom effects size.

Variables	k	Coefficient	*SE*	*p*-value	*R* *^2^*
Session duration	12	-0.72	0.56	0.226	0.00
Total sessions	10	-0.04	0.06	0.493	0.00
Total hours	13	-0.01	0.06	0.936	0.00
Total treatment duration (wk)	13	0.39	0.18	0.052	0.15
Weekly treatment intensity (h/wk)	13	-0.06	0.02	0.017*	0.43

k, number of comparisons; h, hour; wk, week; *p < 0.05, **p < 0.01, ***p < 0.001.

### 3.6 Quality of evidence

Regarding the certainty of evidence at post-treatment, we had very low confidence that intensive CBT outperformed non-active conditions (GRADE: very low). When compared with psychotherapy and pharmacotherapy, we were very uncertain in the effect estimates (GRADE: very low). For the overall pooled estimate across all comparators for OCD, depression, anxiety, and FA, we had very low confidence that intensive CBT outperformed control conditions (GRADE: very low). Additional details are presented in **Supplementary Figures 16**–**17**.

## 4 Discussion

The current work expanded the scope of intensive CBT meta-analysis in three key areas. First, our work includes relatively large sample sizes of 786 patients with OCD based on RCTs, which is larger than those included in some previous meta-analyses (13). Second, intensive CBT showed a large effect when compared with non-active control conditions in the short term. It demonstrated post-treatment efficacy of intensive CBT on reducing OCD symptoms, anxiety, depression, and FA, as well as sustained long-term effects on OCD symptoms. Third, we explored the potential factors influencing treatment effectiveness, identifying weekly treatment intensity as a variable associated with treatment outcome.

### 4.1 Efficacy of intensive CBT on OCD symptoms

The present comprehensive meta-analysis examined the effectiveness of intensive CBT based on fourteen RCTs. Consistent with evidence from standard CBT meta-analyses (6, 7, 65), intensive CBT showed a large effect compared to non-active control conditions in the short term. The effect against non-active controls represents a large effect size. However, its advantage over pharmacotherapy and other active psychological treatments (e.g., standard CBT) did not reach statistical significance. This suggests that while intensive CBT appears superior to minimal interventions, its advantage over other evidence-based treatments is less pronounced, likely because both intensive and standard psychological treatments share common therapeutic mechanisms (e.g., exposure), whereas the non-significant comparison with pharmacotherapy may reflect the limited number of studies in this subgroup.

At follow-up, intensive CBT maintained significant superiority over non-active controls and showed a significant advantage over pharmacotherapy, while its comparison with active psychological treatments remained non-significant. This apparent change in the pharmacotherapy comparison should be interpreted with caution. The follow-up pharmacotherapy subgroup included a different set of studies than the post-treatment analysis, and the number of studies was small, limiting the precision and stability of the estimate. Additionally, differences in maintenance treatment and medication status during follow-up may have contributed to this variation. Therefore, we refrain from interpreting this as evidence that the effect of intensive CBT strengthens over time; rather, it highlights the need for more long-term data with consistent reporting of follow-up procedures and concomitant medication use.

Substantial heterogeneity was detected in each of the three control groups, indicating that this heterogeneity is not solely attributable to control categories. To address this, we conducted moderator analyses and meta-regressions to explore potential sources of variability (35). The funnel plot and Egger’s test suggested small-study effects at post-treatment, one potential explanation for which is publication bias (66). The presence of small-study effects should be acknowledged as a limitation, and the results should be interpreted with this in mind (49, 67).

Subgroup analyses by age revealed that intensive CBT was more effective in adults than in children at follow-up. The test for subgroup differences was significant, suggesting that age moderated the treatment effect in the long term. However, these findings are based on indirect comparisons between different studies rather than head-to-head randomized comparisons of children and adults, and should therefore be interpreted cautiously. Children may find it more challenging to engage in highly concentrated exposure, possibly due to developmental differences in emotional control (68–70). Alternatively, differences in treatment format, family involvement, or comparator type across the child and adult studies may also have influenced the results. Given the limited number of studies in the child subgroup and the indirect nature of the comparison, these findings should be considered hypothesis-generating rather than conclusive.

### 4.2 Efficacy of intensive CBT on secondary outcomes

Intensive CBT showed significant effects on anxiety, depression, and FA reduction at post-treatment. However, at follow-up, only the effect on OCD symptoms remained significant; sustained effects on anxiety, depression, and FA were not demonstrated. This pattern suggests that while intensive CBT produces broad immediate benefits, the durability of its effects on secondary outcomes may be limited, possibly due to the small number of studies with follow-up data, variations in follow-up duration, or the absence of maintenance treatment. Given the limited sample sizes, subgroup analyses were not conducted for secondary outcomes. Moreover, the effect sizes of FA reduction may be influenced by the degree of relatives’ involvement, particularly in studies targeting children (52, 57). Engaging parents could be particularly beneficial when addressing factors linked to poorer CBT outcomes, such as family functioning and symptom accommodation (71).

### 4.3 Factors moderating intensive CBT effects

Exploratory analyses found no significant moderators. Intensive CBT was effective across most subgroups, except for the low-quality and non-medication subgroups where the effect was not significant. However, since the subgroup differences were not significant, these findings should not be interpreted as evidence of moderation; they may suggest potential influences of study quality and combination therapy that warrant further investigation in future RCTs. We did not find significant moderating effects for gender, which differs from previous findings in children with anxiety disorders (12). A plausible explanation is that the relatively small number of studies in subgroup analyses may have limited power to detect such effects. Given that numerous clinical studies have demonstrated the effectiveness of intensive CBT for OCD, most of which were single-arm trials or non-randomized studies (17, 18, 72, 73), the importance of considering study quality when interpreting pooled estimates is underscored.

Turning to meta-regression, we coded five variables related to time intensity, as referenced in previous meta-analyses (12, 13). Among these, we found that weekly treatment intensity was significantly associated with effect size, suggesting that higher weekly intensity may be associated with slightly larger effect sizes. However, given the exploratory nature of this analysis and the small number of studies, this finding should be interpreted as hypothesis-generating rather than causal. We also acknowledge that the association may be confounded by other study-level factors, such as comparator type, treatment format, or age. Other intensity indicators, including session duration, total sessions, total hours, and total treatment duration did not reach statistical significance. Our findings partially align with Öst and Ollendick (12), who found that intensity (hours/week) significantly moderated outcomes while total hours did not, supporting the notion that treatment frequency and intensity may be more relevant than total therapy duration in intensive CBT. In contrast, Jónsson, Kristensen and Arendt (13) found that total treatment hours were significantly associated with the effect sizes in their research. These discrepancies may be attributed to sample heterogeneity, as some prior meta-analyses included both anxiety disorders and OCD or focused exclusively on children, whereas our study included both children and adults with OCD. Another possible reason is that some studies included non-RCTs and did not account for baseline-posttest correlations (27). Given the limited number of studies contributing to these analyses, these findings should be interpreted as exploratory. We cannot exclude the possibility that the observed association between weekly intensity and treatment effect is confounded by other study-level factors, such as comparator type or treatment format. Therefore, the differences between our study and previous research should be interpreted with caution.

### 4.4 Limitations

Several limitations should be considered. First, the definition of intensive CBT remains relatively broad, encompassing variations in format, duration, and therapeutic components (e.g., ERP-focused vs mixed approaches, with or without concomitant pharmacotherapy). This heterogeneity may introduce conceptual ambiguity and reduce the interpretability of pooled estimates. Some studies allowed concurrent medication use, implying that a proportion of patients received combination therapy. As combination treatment has been proven more effective than psychological treatment or pharmacotherapy alone, we need to be cautious in estimating subgroup analyses of control conditions (74). In addition, we excluded remotely delivered CBT to maintain homogeneity in treatment delivery format. While we acknowledge that remote delivery may have implications for accessibility, the limited number of studies and the potential increase in heterogeneity precluded their inclusion. Future research on remote intensive CBT formats is warranted. Second, the combination of heterogeneous comparator conditions (non-active controls, pharmacotherapy, and active psychological treatments) within the main analyses complicates the interpretation of the overall effect size. To address this, we performed stratified analyses by comparator type and did not report an overall pooled effect size across all comparators. Third, the follow-up duration varied widely, ranging from two months to 17.5 months, making it challenging to compare effect sizes across studies (54, 60, 61). Fourth, maintenance treatment varied widely. Foa et al. (55) provided weekly 45-minute sessions for 8 weeks after intensive ERP, nearly reaching the standard of weekly CBT, while Vogel et al. (61) offered monthly 15-minute telephone follow-ups. These differences in maintenance treatment could lead to significant variations in efficacy evaluations at follow-up. Fifth, publication bias assessment for follow-up analyses was limited by the small number of studies (k = 8). At follow-up, although the funnel plot appeared symmetrical, the fail-safe N was small, suggesting limited robustness. This suggests that the follow-up findings should be interpreted with caution and underscores the need for more long-term data. Finally, the GRADE assessment indicated very low certainty for all post-treatment comparisons and low certainty for follow-up non-active and pharmacotherapy comparisons. Although the follow-up comparisons were rated slightly higher due to narrower confidence intervals and less heterogeneity, this should be interpreted with caution: the small number of studies, wide variation in follow-up duration, heterogeneous maintenance treatments, and incomplete medication reporting may limit clinical interpretability, even though these factors are not direct GRADE downgrading criteria. This low to very low certainty should be prominently reflected in the framing of conclusions. Increasing sample sizes and improving trial methodological quality would help to reduce heterogeneity and improve the precision of future estimates.

Our paper highlights the need for future research in several areas. First, the definition of intensive CBT needs to be standardized. Even though we set criteria for study identification based on an extensive literature review, researchers and clinicians must reach a consensus on treatment intensity. Second, high methodological quality is critical for convincing meta-analysis conclusions. RCTs with low risk of bias are required to upgrade the GRADE recommendation level. Third, the low to very low certainty of evidence (GRADE) underscores the need for more rigorous trials with consistent reporting of follow-up procedures and maintenance treatment. Fourth, other outcomes such as response, remission, dropout, treatment completion, and adverse events are important for evaluating clinical usefulness but were not covered in the current study and warrant further investigation. Finally, given the limited long-term data, future trials should systematically report follow-up outcomes to better establish the durability of intensive CBT effects.

## Data Availability

The original contributions presented in the study are included in the article/**Supplementary Material**. Further inquiries can be directed to the corresponding author.
