# Supplementary material for: Intensive cognitive behavioral therapy for obsessive-compulsive disorder: a meta-analysis of randomized controlled trials
Source: Front Psychiatry. 2026 Sep 28;17:1937285. doi: 10.3389/fpsyt.2026.1937285 (PMC13655579; doi:10.3389/fpsyt.2026.1937285)
Supplement: Supplementary file 1 [file DataSheet1.pdf]

Supplementary Material

1 Supplementary Figures and Tables

1.1 Supplementary Table

Supplementary Table 1. Search strategy for each database.

Database (Platform)	Search strategy	Filters	Records
PubMed (PubMed.gov)	(obsess* OR compul*) AND ((cognitive behavior* AND (therap* OR psychotherap* OR treatment OR intervention)) OR exposure response prevention) AND (intensive* or concentrate* or short* or short-term or week* or day or daily or quick*) AND (random* OR control* OR placebo* OR blind*)	None (default settings)	810
Cochrane (Wiley Online Library)	(obsess* OR compul*) AND ((cognitive behavior* AND (therap* OR psychotherap* OR treatment OR intervention)) OR exposure response prevention) AND (intensive* or concentrate* or short* or short-term or week* or day or daily or quick*) AND (random* OR control* OR placebo* OR blind*)	None (default settings)	786
Embase (Elsevier)	((obsess* OR compul*) AND (cognitive behavior* AND (therap* OR psychotherap* OR treatment OR intervention) OR exposure response prevention) AND (intensive* or concentrate* or short* or short-term or week* or day or daily or quick*) AND (random* OR control* OR placebo* OR blind*))	None (default settings)	1611
Medline (EBSCOhost)	(obsess* OR compul*) AND ((cognitive behavior* AND (therap* OR psychotherap* OR treatment OR intervention)) OR exposure response prevention) AND (intensive* or concentrate* or short* or short-term or week* or day or daily or quick*) AND (random* OR control* OR placebo* OR blind*)	None (default settings)	967
PsycINFO (EBSCOhost)	(obsess* OR compul*) AND ((cognitive behavior* AND (therap* OR psychotherap* OR treatment OR intervention)) OR exposure response prevention) AND (intensive* or concentrate* or short* or short-term or week* or day or daily or quick*) AND (random* OR control* OR placebo* OR blind*)	None (default settings)	602
Web of Science	(obsess* OR compul*) AND ((cognitive behavior* AND (therap* OR psychotherap* OR treatment OR intervention)) OR exposure response prevention) AND (intensive* or concentrate* or short* or short-term	None (default	985

(Clarivate)	or week* or day or daily or quick*) AND (random* OR control* OR placebo* OR blind*)	settings)	
CNKI	（主题：强迫 + 强迫症 + 强迫障碍 + 强迫状态）AND（篇关摘：短程 + 强化 + 集中 + 密集 + 简短 + 快捷 + 4 周 + 四周（精确））AND（篇关摘：认知行为 + 认知 + 行为 + 暴露）AND（篇关摘：疗法 + 治疗 + 干预（精确））AND（篇关摘：随机 + 对照 + 控制 + 盲法 + 安慰剂）	None (default settings; Chinese)	239
WanFang	(强迫 OR 强迫症 OR 强迫障碍 OR 强迫状态) and 全部:(认知行为 OR 认知 OR 行为 OR 暴露) and 全部:(疗法 OR 治疗 OR 干预) and 全部:(短程 OR 强化 OR 集中 OR 密集 OR 简短 OR 快捷 OR 4 周 OR 四周) and 全部:(随机 OR 对照 OR 控制 OR 盲法 OR 安慰剂)	None (default settings; Chinese)	313

Supplementary Table 2. Characteristics of randomized controlled trials included in the meta-analysis.

Study	Compare groups	Duration of each session (h/session)	Total sessions	Total hours(h)	Total Duration	Total sample	Mean age(SD)	sample	%Female	Medication	%comorbidity	Follow-up (maximum)	Outcome measures of OCD symptoms	Jadad score
Barros, et al., 2024 (51)	1) Intenisve CBT<br>2) Waiting list	1.5	14	20h	4wk	30	13.00(2.77)[a]<br>12.57(2.47)[b]	Child	46.67	No	23%	6mth	CY-BOCS	5
Baruah et al., 2018 (52)	1) Intensive CBT<br>2) Relaxation exercises	1.5-2	6	12h	3-4wk	64	30.40(7.96)[a]<br>30.64(7.66)[b]	Adult	54.69	Yes	37%	3mth	Y-BOCS	5
Bolton et al., 2008 (53)	1) Intensive CBT<br>2) Waiting list	1-1.5	10	15h	5wk	20	13.20(2.33)[a]<br>13.42(2.42)[b]	Child	30.00	No	50%	3.5mth	CY-BOCS	4
Challacombe et al., 2017 (54)	1) Intensive CBT<br>2) TAU	3	4	12	2wk	34	26.82(9.96)[a]<br>24.18(6.20)[b]	Adult	100%	Yes	24%	5.5mth	Y-BOCS	4
Foa et al.,	1) Intensive ERP	2	15	30	3wk	149	33.8(8.9)[a]	Adult	48.00	1) No	0	3mth	Y-BOCS	5

2005 (55)	2) Itensive ERP + Clomipramine<br>3) Clomipramine<br>4) Placebo						35.0(12.2)[a]<br>35.7(11.3)[b]<br>34.3(11.4)[b]			2) Yes				
Launes et al., 2019 (56)	1) Intensive CBT<br>2) Wait-list<br>3) Self-help	1.5	NR	24h	4d	48	30.35(11.08)	Adult	79.00	Yes	87%	6mth	Y-BOCS	4
Lewin et al., 2014 (57)	1) Intensive CBT<br>2) TAU	1	12	12h	6wk	31	5.76(1.52)[a]<br>5.86(1.70)[b]	Child	29.03	Yes	71%	3mth	CY-BOCS	5
Lindsay et al., 1997 (58)	1) Intensive CBT<br>2) Anxiety management	NR	NR	15h	3wk	18	31.6(8.9)[a]<br>34.0(9.3)[b]	Adult	66.67	Yes	NR	NR	Y-BOCS	3
Abramowitz et al., 2003 (59)	1) Intensive CBT<br>2) Weekly CBT	2	15	30h	3wk	40	36.2(15.6)[a]<br>38.7(13.6)[b]	Adult	42.50	Yes	45%	3mth	Y-BOCS	3
Cottraux et al., 2001 (60)	1) Intensive CBT<br>2) CT	2	8	16h	4wk	64	25.75(3.9)[a]<br>25.55(4.5)[b]	Adult	74.19	No	43%	12mth	Y-BOCS	2
Storch et al., 2007 (10)	1) Intensive CBT<br>2) Standard CBT	1.5	14	21h	3wk	40	13.3(2.7)[a]<br>14.5(2.8)[b]	Child	55.00	Yes	75%	3mth	CY-BOCS	3
Vogel et al., 2004 (61)	1) Intensive CBT<br>2) ERP+relaxation training	2	12	24h	6wk	35	35.7(12.1)	Adult	28.57	Yes	100%	12mth	Y-BOCS	4
Kayser et al., 2020 (62)	1) Intensive ERP	1.5	9	13.5h	4wk	11	30.8(10.4)[a]	Adult	54.54	Yes	18%	2mth	Y-BOCS	4

	2) Nabilone						35.5(14.6)[b]							
	1) Intensive CBT						25.3(7.6)[a]							
Xiao et al., 2019 (63)	2) SSRIs, SNRIs	2	8	16h	4wk	202	23.2(8.9)[b]	Adult	50.00	Yes	0	NR	Y-BOCS	3

Note. Drop-out = numbers of drop-outs at post-treatment. M = mean, SD = standard deviation, a = intensive group, b = control group. %F = the percentage of females. CBT = cognitive behavioral therapy, h = hour, wk = week, mth = month. NR = no reported. CT = cognitive therapy. ERP = exposure and response prevention. TAU = treatment as usual. SRIs = selective serotonin reuptake inhibitors, SSRIs = selective serotonin re-uptake inhibitors, SNRIs = serotonin and norepinephrine reuptake inhibitors. Y-BOCS = Yale-Brown Obsessive Compulsive Scale, CY-BOCS = Children Yale-Brown Obsessive Compulsive Scale, MOCI = Maudsley Obsessional-Compulsive Inventory, ASR = Assessor Severity Rating.

Supplementary Table 3. Detailed reasons for full-text exclusion.

Excluded study	Title	Detailed reason
Tjelle et al., 2025	Concentrated ERP for Patients With Difficult-to-Treat OCD: Insight as a Predictor of Acute and Long-Term Outcomes	Secondary analysis.
Berg et al., 2025	Treatment expectancy and credibility as predictors of concentrated exposure treatment outcomes in patients with difficult-to-treat obsessive-compulsive disorder	Secondary analysis.
Jaurrieta et al., 2024	Challenges in testing concentrated cognitive-behaviour therapy for unmedicated adults with obsessive-compulsive disorder in routine care	Insufficient data: study terminated early due to recruitment difficulties; not a research article.
Jelinek et al., 2024	Exploring the Peaks and Potholes: Understanding positive and negative effects of concentrated exposure treatment for obsessive-compulsive disorder	Not an RCT: compared with matched historical inpatient control group.
Challacombe et al., 2024	Exposure-based cognitive-behaviour therapy for anxiety-related disorders in pregnancy (ADEPT): Results of a feasibility randomised controlled trial of time-intensive versus weekly CBT	Insufficient data: OCD data pooled with other anxiety disorder groups; separate OCD data unavailable.
Landrø et al., 2024	Effect of concentrated exposure and response prevention on symptoms of insomnia	Aims not fitting: all participants received B4DT; no non-CBT control group for

		comparison.
Thorsen et al., 2020	Stable inhibition-related inferior frontal hypoactivation and fronto-limbic hyperconnectivity in obsessive-compulsive disorder after concentrated exposure therapy	Aims not fitting: RCT comparing OCD patients with healthy controls; not evaluating treatment efficacy.
Kvale et al., 2020	Effect of D-Cycloserine on the Effect of Concentrated Exposure and Response Prevention in Difficult-to-Treat Obsessive-Compulsive Disorder	Aims not fitting: all participants received B4DT combined with DCS or placebo; no non-CBT control group.
Olatunji et al., 2013	Behavioral Versus Cognitive Treatment of Obsessive-Compulsive Disorder: An Examination of Outcome and Mediators of Change	Secondary analysis.
Storch et al., 2010	Defining Treatment Response and Remission in Obsessive-Compulsive Disorder: A Signal Detection Analysis of the Children's Yale-Brown Obsessive Compulsive Scale	Secondary analysis.
Saxena et al., 2009	Rapid effects of brief intensive cognitive-behavioral therapy on brain glucose metabolism in obsessive-compulsive disorder	Aims not fitting: RCT comparing OCD patients with healthy controls.
Hembree et al., 2003	Long-Term Efficacy of Exposure and Ritual Prevention Therapy and Serotonergic Medications for Obsessive-Compulsive Disorder	Not an RCT: long-term follow-up of an RCT.
Franklin et al., 1998	Cognitive-Behavioral Treatment of Pediatric Obsessive-Compulsive Disorder: An Open Clinical Trial	Insufficient data; open trial without control group.

1.2 Supplementary Figures

Study name	Statistics with study removed						
	Point	Standard error	Variance	Lower limit	Upper limit	Z-Value	p-Value
Abramowitz, 2003	-1.136	0.256	0.065	-1.637	-0.635	-4.444	0.000
Barros, 2024	-1.050	0.246	0.060	-1.532	-0.569	-4.274	0.000
Baruah, 2018	-1.152	0.262	0.069	-1.665	-0.639	-4.400	0.000
Bolton, 2008	-1.096	0.248	0.062	-1.583	-0.609	-4.413	0.000
Cottraux, 2001	-1.188	0.247	0.061	-1.672	-0.703	-4.804	0.000
Kayser, 2020	-1.037	0.240	0.058	-1.508	-0.567	-4.321	0.000
Launes, 2019a	-0.869	0.204	0.042	-1.269	-0.468	-4.252	0.000
Launes, 2019b	-0.955	0.228	0.052	-1.401	-0.509	-4.194	0.000
Lewin, 2014	-1.012	0.239	0.057	-1.481	-0.543	-4.231	0.000
Lindsay, 1997	-0.934	0.222	0.049	-1.369	-0.498	-4.202	0.000
Storch, 2007	-1.145	0.255	0.065	-1.645	-0.645	-4.490	0.000
Vogel, 2004	-1.179	0.246	0.061	-1.662	-0.696	-4.784	0.000
Xiao, 2019	-1.180	0.283	0.080	-1.734	-0.626	-4.172	0.000
	-1.068	0.233	0.054	-1.525	-0.611	-4.580	0.000

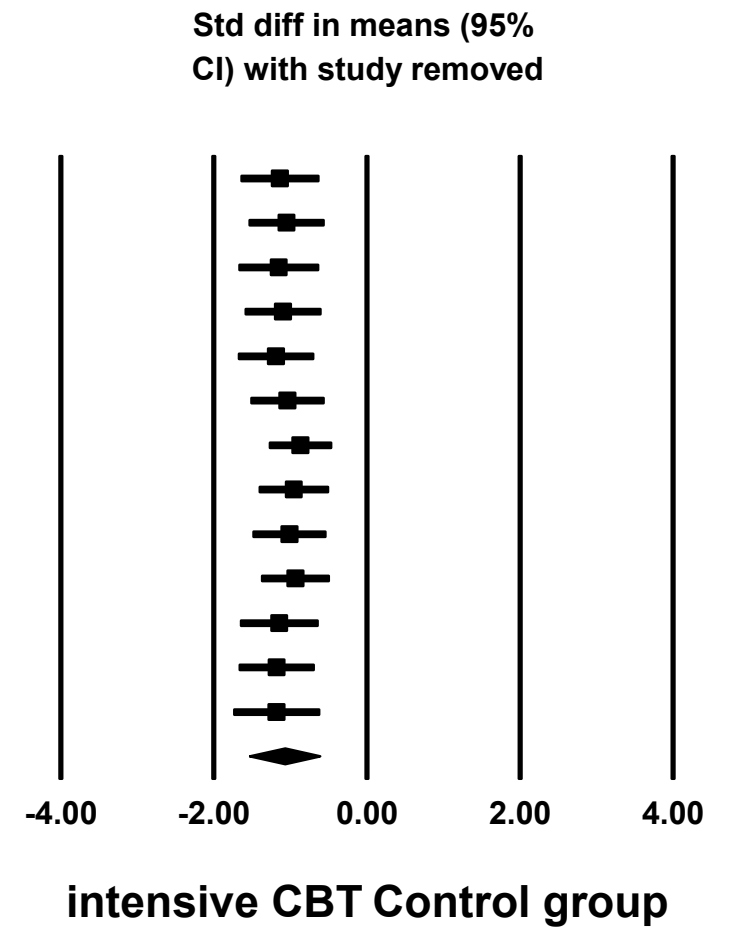

Supplementary Figure 1. Sensitivity analysis for the effects of intensive CBT vs. control groups on OCD symptoms at post-treatment.

Study name	Statistics with study removed						
	Point	Standard error	Variance	Lower limit	Upper limit	Z-Value	p-Value
Barros, 2024	-1.321	0.343	0.117	-1.993	-0.650	-3.857	0.000
Baruah, 2018	-1.474	0.374	0.140	-2.207	-0.740	-3.939	0.000
Launes, 2019a	-1.034	0.272	0.074	-1.567	-0.501	-3.803	0.000
Launes, 2019b	-1.172	0.311	0.097	-1.782	-0.561	-3.763	0.000
Lewin, 2014	-1.267	0.332	0.110	-1.918	-0.615	-3.811	0.000
Lindsay, 1997	-1.131	0.301	0.091	-1.721	-0.540	-3.755	0.000
Storch, 2007	-1.461	0.358	0.128	-2.163	-0.760	-4.082	0.000
Vogel, 2004	-1.510	0.340	0.116	-2.177	-0.844	-4.441	0.000
Xiao, 2019	-1.499	0.398	0.159	-2.279	-0.718	-3.763	0.000
	-1.311	0.312	0.097	-1.923	-0.699	-4.198	0.000

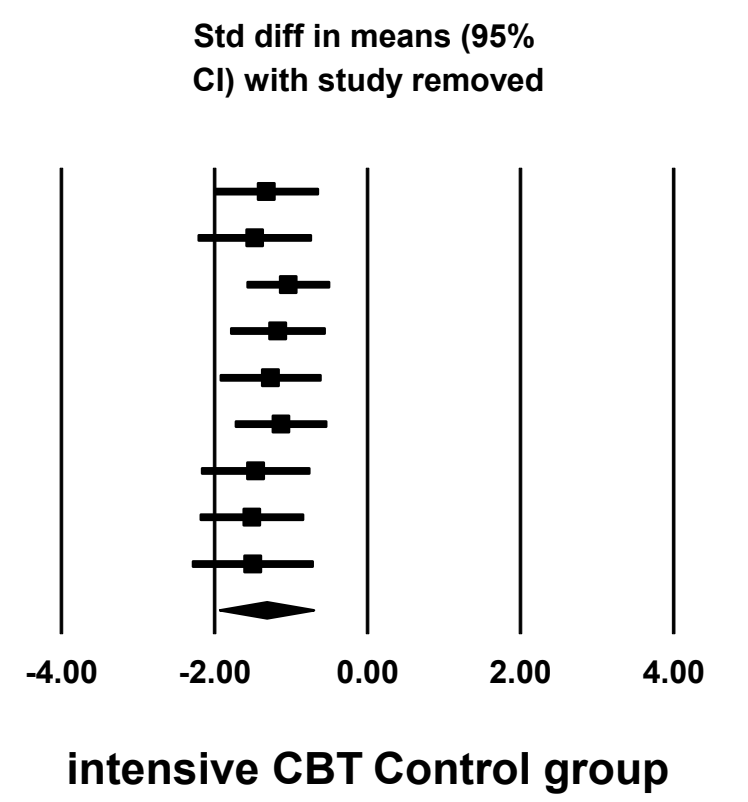

Supplementary Figure 2. Sensitivity analysis excluding high-risk studies at post-treatment.

Study name	Point	Standard error	Variance	Lower limit	Upper limit	Z-Value	p-Value
	Statistics with study removed						
Abramowitz, 2003	-1.061	0.264	0.070	-1.579	-0.543	-4.012	0.000
Barros, 2024	-0.972	0.253	0.064	-1.468	-0.475	-3.837	0.000
Baruah, 2018	-1.077	0.272	0.074	-1.609	-0.545	-3.967	0.000
Bolton, 2008	-1.020	0.256	0.066	-1.523	-0.518	-3.979	0.000
Cottraux, 2001	-1.119	0.257	0.066	-1.622	-0.616	-4.361	0.000
Kayser, 2020	-0.961	0.247	0.061	-1.446	-0.476	-3.885	0.000
Launes, 2019a	-0.737	0.190	0.036	-1.109	-0.364	-3.873	0.000
Lewin, 2014	-0.933	0.246	0.061	-1.415	-0.450	-3.790	0.000
Lindsay, 1997	-0.856	0.228	0.052	-1.302	-0.409	-3.757	0.000
Storch, 2007	-1.071	0.264	0.070	-1.589	-0.554	-4.057	0.000
Vogel, 2004	-1.110	0.255	0.065	-1.610	-0.610	-4.349	0.000
Xiao, 2019	-1.103	0.295	0.087	-1.681	-0.525	-3.738	0.000
	-0.998	0.240	0.058	-1.469	-0.528	-4.159	0.000

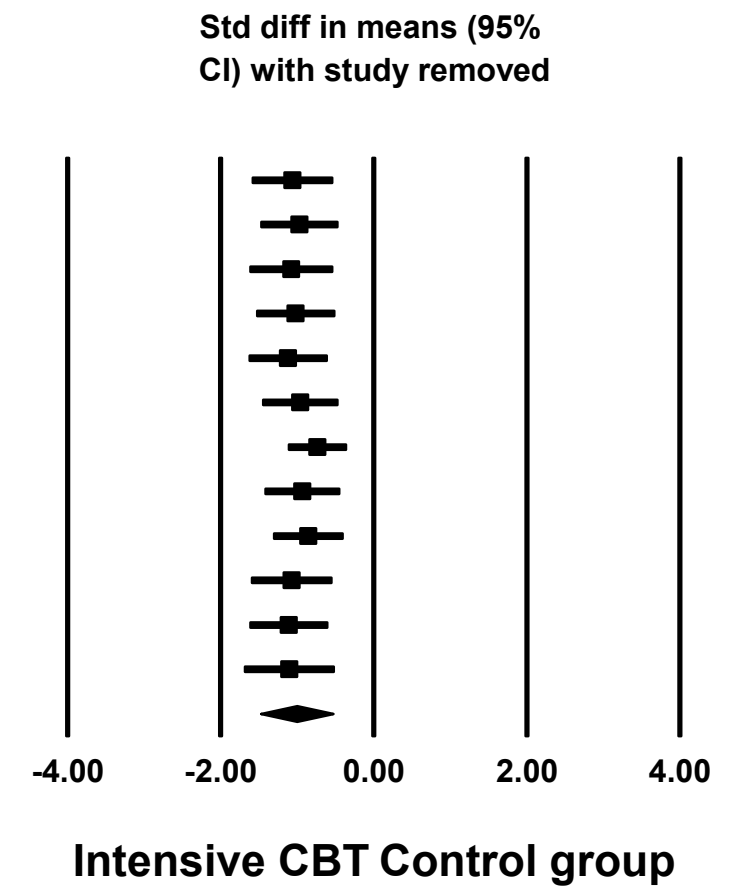

Supplementary Figure 3. Sensitivity analysis including independent comparisons per multi-arm study at post-treatment.

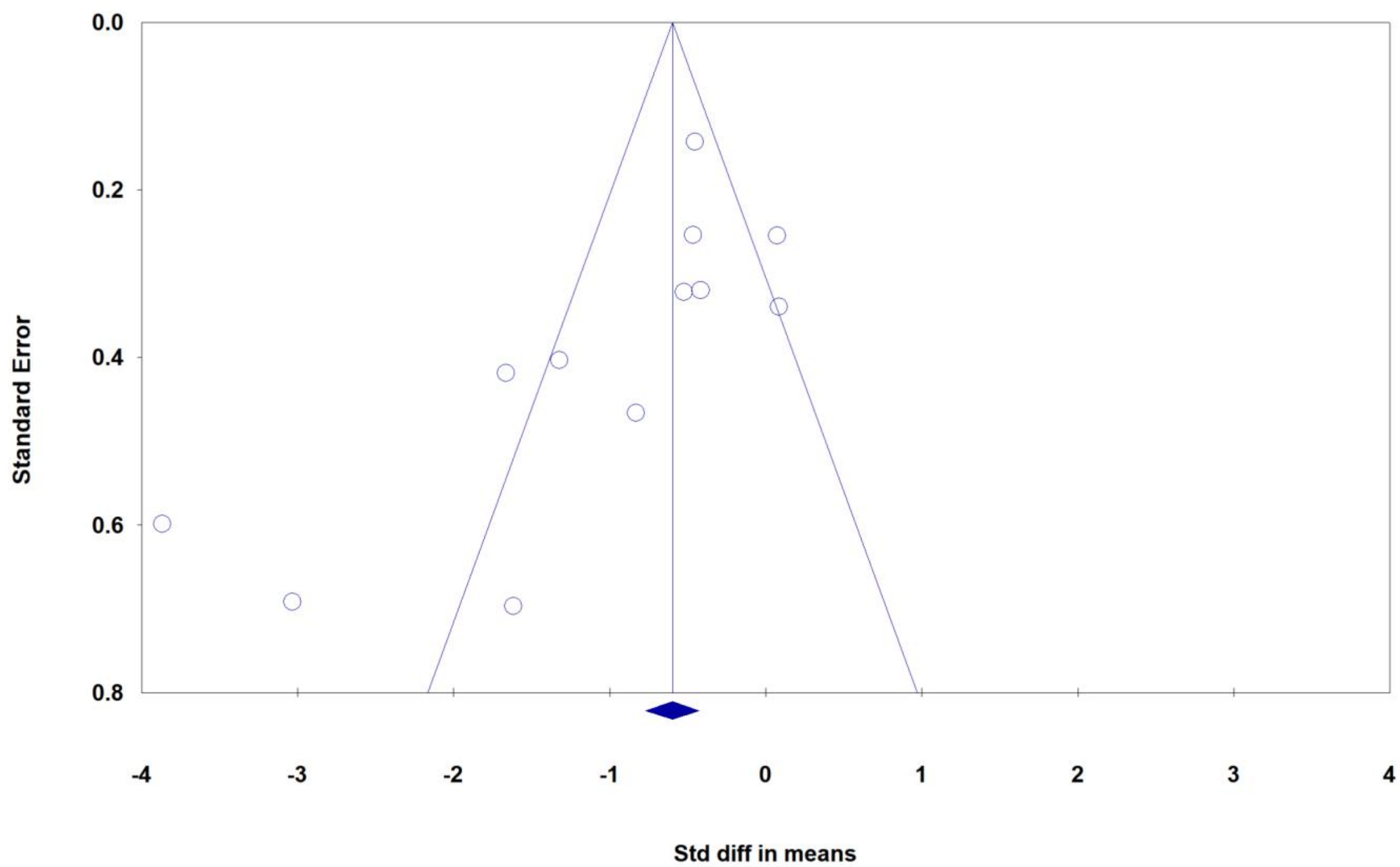

Supplementary Figure 4. Publication bias analysis for the effects of intensive CBT on OCD symptoms at post-treatment.

Study or Subgroup	Intensive CBT Mean	Intensive CBT SD	Intensive CBT Total	Control Mean	Control SD	Control Total	Weight	Std. Mean Difference IV, Random, 95% CI
**1.6.1 Child**								
Barros, 2024	17.75	6.53	15	25.5	5.1	15	7.9%	-1.29 [-2.08, -0.49]
Bolton, 2008	13.9	10.74	10	21.1	5.9	10	7.3%	-0.80 [-1.71, 0.12]
Lewin, 2014	12.29	8.61	17	24	4.35	14	7.8%	-1.62 [-2.45, -0.79]
Storch, 2007	9.5	6.9	20	12.8	8.8	20	8.8%	-0.41 [-1.04, 0.22]
**Subtotal (95% CI)**			**62**			**59**	**31.9%**	**-1.00 [-1.56, -0.43]**
Heterogeneity: Tau² = 0.17; Chi² = 6.19, df = 3 (P = 0.10); I² = 51%								
Test for overall effect: Z = 3.47 (P = 0.0005)								
**1.6.2 Adult**								
Abramowitz, 2003	11.8	6.2	20	15.3	7.1	20	8.8%	-0.51 [-1.15, 0.12]
Baruah, 2018	20.2	5.81	30	22.64	4.69	34	9.5%	-0.46 [-0.96, 0.04]
Cottraux, 2001	8.5	6.7	32	8	7.2	30	9.5%	0.07 [-0.43, 0.57]
Kayser, 2020	14.2	4.8	5	24	6.9	6	5.0%	-1.48 [-2.89, -0.06]
Launes, 2019a	10.9	4.35	8	27.32	4.14	16	4.9%	-3.77 [-5.21, -2.33]
Launes, 2019b	10.9	4.35	8	24.63	6.18	16	6.3%	-2.34 [-3.46, -1.23]
Lindsay, 1997	11	3.81	9	25.89	5.8	9	5.0%	-2.89 [-4.30, -1.48]
Vogel, 2004	16.7	7.2	16	16.1	7.3	19	8.6%	0.08 [-0.58, 0.75]
Xiao, 2019	22.3	4.4	101	24.3	4.4	101	10.4%	-0.45 [-0.73, -0.17]
**Subtotal (95% CI)**			**229**			**251**	**68.1%**	**-1.03 [-1.59, -0.46]**
Heterogeneity: Tau² = 0.55; Chi² = 51.11, df = 8 (P < 0.00001); I² = 84%								
Test for overall effect: Z = 3.56 (P = 0.0004)								
**Total (95% CI)**			**291**			**310**	**100.0%**	**-1.00 [-1.43, -0.56]**
Heterogeneity: Tau² = 0.45; Chi² = 61.14, df = 12 (P < 0.00001); I² = 80%								
Test for overall effect: Z = 4.51 (P < 0.00001)								
Test for subgroup differences: Chi² = 0.01, df = 1 (P = 0.94), I² = 0%								

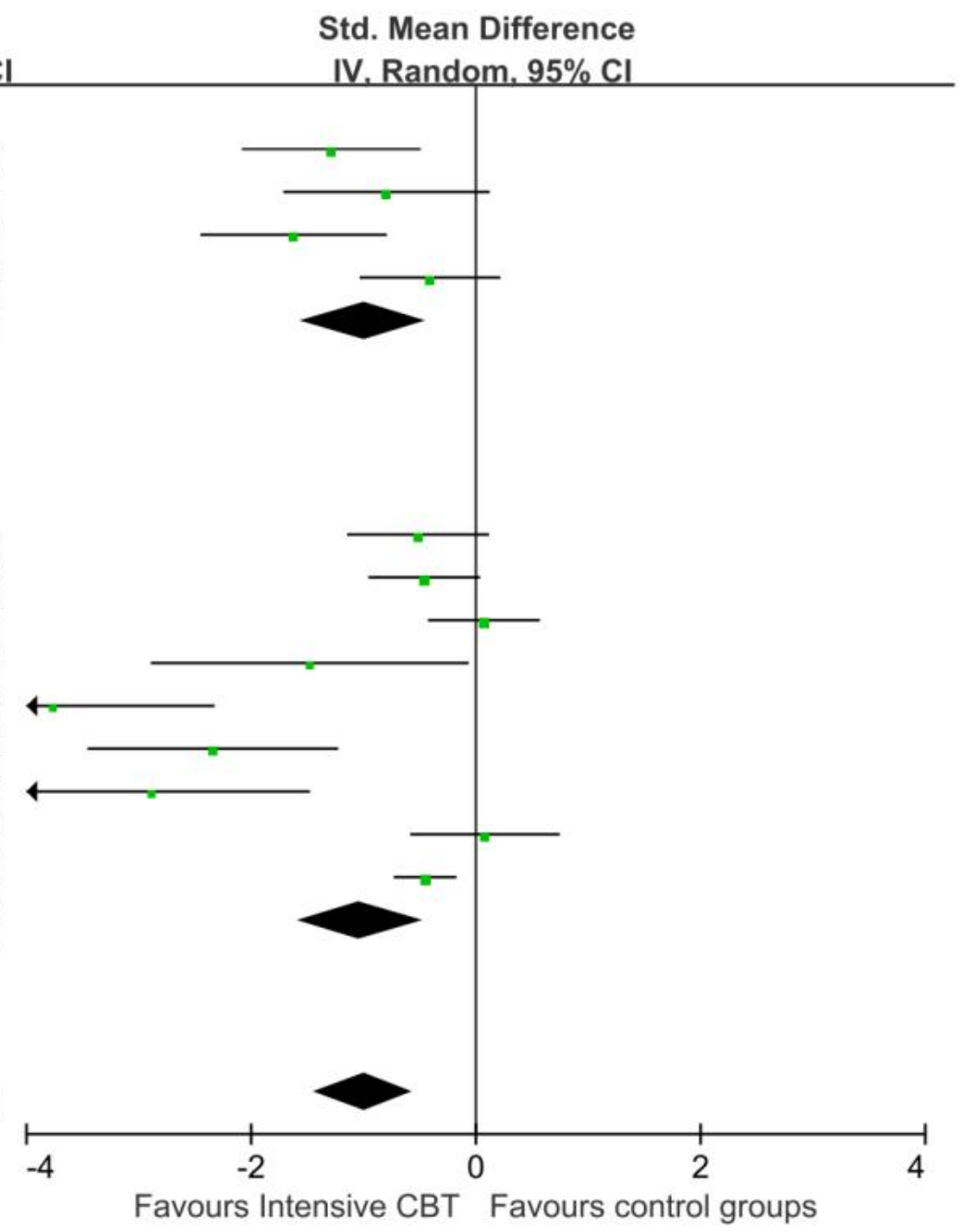

Supplementary Figure 5. Subgroup analysis of intensive CBT effects on OCD symptoms by age group (children vs. adults) at post-treatment.

Study name	Point	Standard error	Variance	Lower limit	Upper limit	Z-Value	p-Value
Abramowitz, 2003	-0.695	0.226	0.051	-1.137	-0.252	-3.079	0.002
Baruah, 2018	-0.636	0.233	0.054	-1.093	-0.179	-2.728	0.006
Challacombe, 2017	-0.608	0.221	0.049	-1.041	-0.174	-2.748	0.006
Cottraux, 2001	-0.737	0.183	0.033	-1.095	-0.379	-4.034	0.000
Kayser, 2020	-0.571	0.205	0.042	-0.972	-0.170	-2.789	0.005
Storch, 2007	-0.708	0.217	0.047	-1.135	-0.282	-3.257	0.001
Vogel, 2004	-0.701	0.222	0.049	-1.136	-0.266	-3.160	0.002
Foa, 2005a	-0.550	0.203	0.041	-0.948	-0.152	-2.708	0.007
Foa, 2005b	-0.610	0.219	0.048	-1.040	-0.180	-2.781	0.005
Foa, 2005c	-0.549	0.202	0.041	-0.945	-0.152	-2.711	0.007
Foa, 2005d	-0.605	0.218	0.048	-1.033	-0.178	-2.775	0.006
	-0.633	0.204	0.042	-1.032	-0.233	-3.104	0.002

(Statistics with study removed)

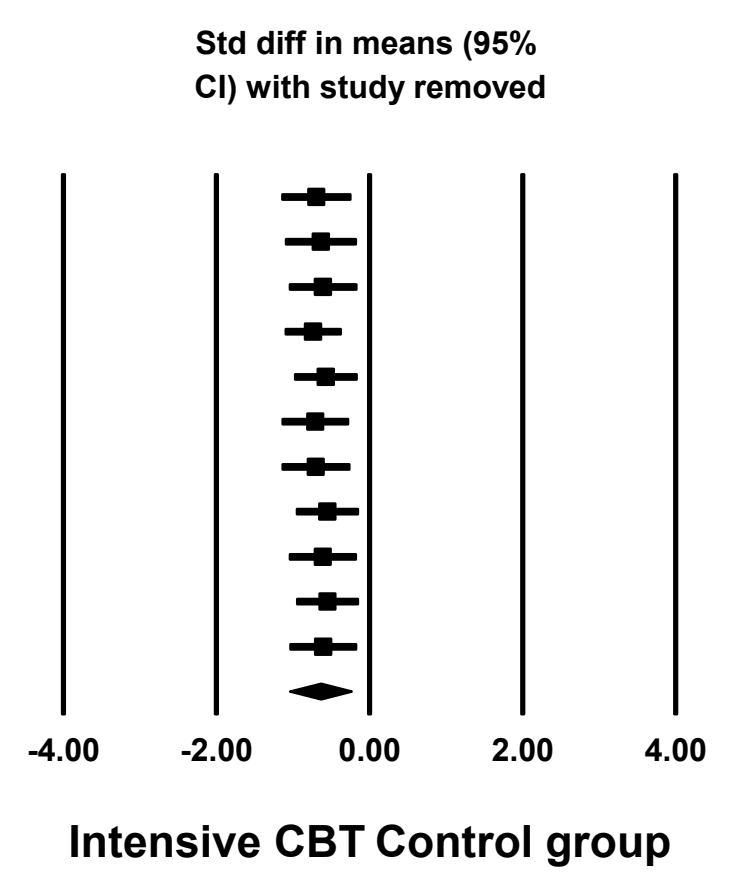

Supplementary Figure 6. Sensitivity analysis for the effects of intensive CBT vs. control groups on OCD symptoms at follow-up.

Study name	Point	Standard error	Variance	Lower limit	Upper limit	Z-Value	p-Value
	Statistics with study removed						
Abramowitz, 2003	-0.614	0.254	0.065	-1.112	-0.116	-2.416	0.016
Baruah, 2018	-0.542	0.263	0.069	-1.058	-0.026	-2.059	0.039
Challacombe, 2017	-0.515	0.248	0.062	-1.002	-0.029	-2.075	0.038
Cottraux, 2001	-0.686	0.206	0.042	-1.090	-0.283	-3.333	0.001
Kayser, 2020	-0.482	0.228	0.052	-0.929	-0.034	-2.110	0.035
Storch, 2007	-0.634	0.244	0.060	-1.114	-0.155	-2.596	0.009
Vogel, 2004	-0.624	0.250	0.062	-1.113	-0.134	-2.498	0.012
Foa, 2005a	-0.421	0.212	0.045	-0.835	-0.006	-1.988	0.047
Foa, 2005d	-0.505	0.249	0.062	-0.992	-0.018	-2.032	0.042
	-0.557	0.226	0.051	-1.000	-0.114	-2.465	0.014

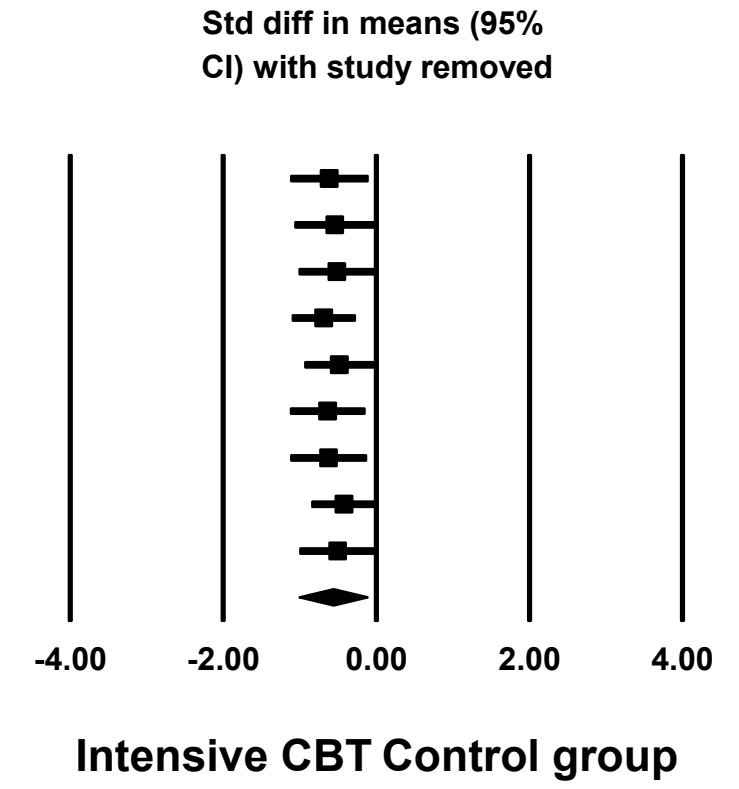

Supplementary Figure 7. Sensitivity analysis including independent comparisons per multi-arm study at follow-up.

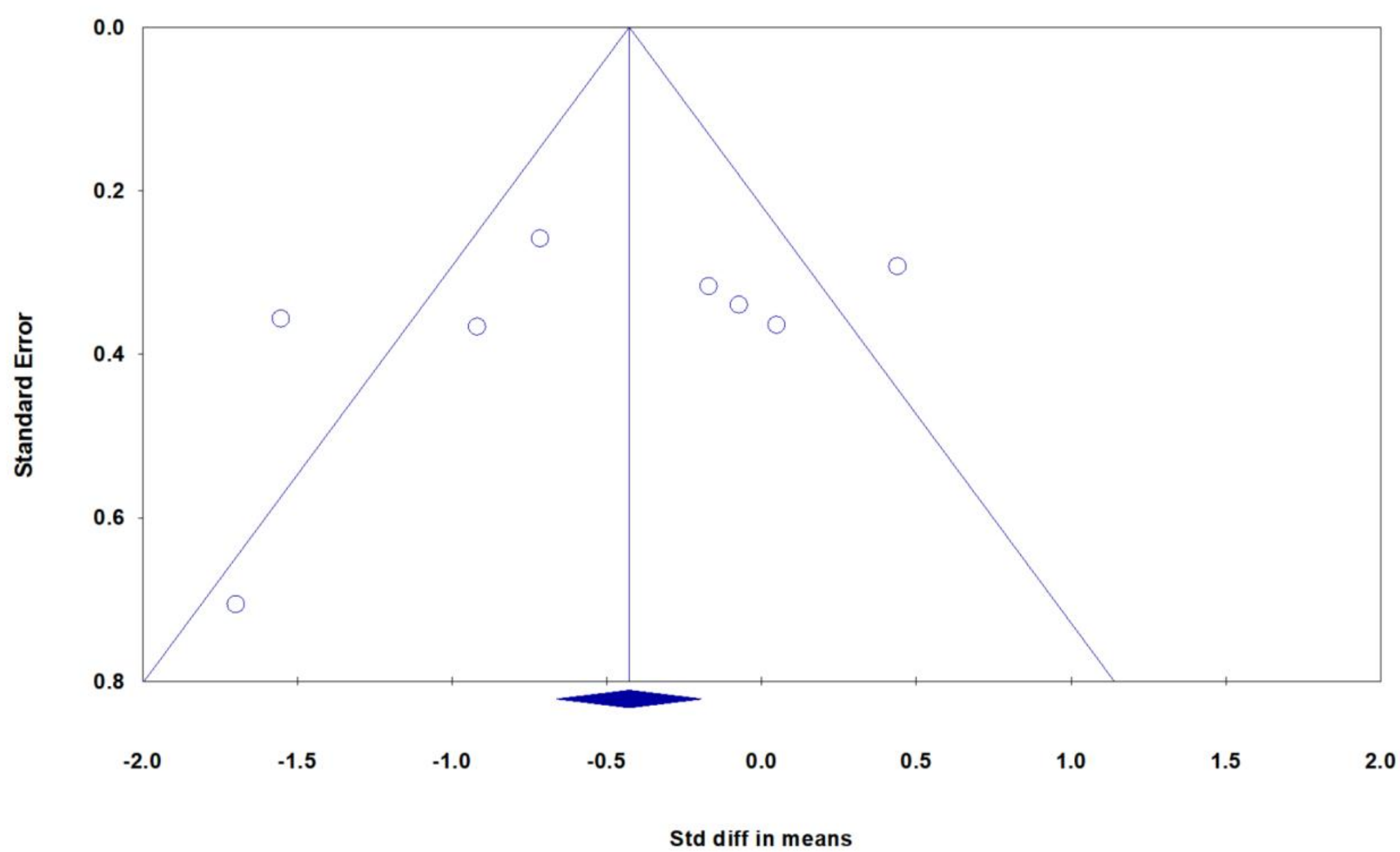

Supplementary Figure 8. Publication bias analysis for the effects of intensive CBT on OCD symptoms at follow-up.

Study or Subgroup	intensive CBT Mean	SD	Total	Control condition Mean	SD	Total	Weight	Std. Mean Difference IV, Random, 95% CI
**2.2.1 Child**								
Storch, 2007	10.2	8.7	18	9.8	7.6	13	9.8%	0.05 [-0.67, 0.76]
Vogel, 2004	14.8	8.3	16	15.4	8.1	19	10.2%	-0.07 [-0.74, 0.59]
**Subtotal (95% CI)**			**34**			**32**	**20.0%**	**-0.02 [-0.50, 0.47]**
Heterogeneity: Tau² = 0.00; Chi² = 0.06, df = 1 (P = 0.81); I² = 0%								
Test for overall effect: Z = 0.07 (P = 0.95)								
**2.2.2 Adult**								
Abramowitz, 2003	13.75	5.4	20	14.7	5.7	20	10.7%	-0.17 [-0.79, 0.45]
Baruah, 2018	17.13	7.04	30	21.76	5.92	34	11.8%	-0.71 [-1.21, -0.20]
Challacombe, 2017	13.71	8.95	17	20.88	6.34	16	9.7%	-0.90 [-1.62, -0.18]
Cottraux, 2001	17.4	8.5	23	13.6	8.8	25	11.1%	0.43 [-0.14, 1.01]
Foa, 2005a	11	7.9	11	22.2	6.4	10	7.4%	-1.49 [-2.48, -0.50]
Foa, 2005b	11	7.9	11	18.2	7.8	14	8.7%	-0.89 [-1.72, -0.05]
Foa, 2005c	10.5	8.2	10	22.2	6.4	10	7.2%	-1.52 [-2.55, -0.50]
Foa, 2005d	10.5	8.2	10	18.2	7.8	14	8.5%	-0.93 [-1.79, -0.07]
Kayser, 2020	15.8	4.6	5	24.5	5.5	6	4.8%	-1.55 [-2.99, -0.12]
**Subtotal (95% CI)**			**137**			**149**	**80.0%**	**-0.75 [-1.20, -0.30]**
Heterogeneity: Tau² = 0.29; Chi² = 24.05, df = 8 (P = 0.002); I² = 67%								
Test for overall effect: Z = 3.28 (P = 0.001)								
**Total (95% CI)**			**171**			**181**	**100.0%**	**-0.59 [-0.98, -0.21]**
Heterogeneity: Tau² = 0.26; Chi² = 28.38, df = 10 (P = 0.002); I² = 65%								
Test for overall effect: Z = 3.04 (P = 0.002)								
Test for subgroup differences: Chi² = 4.72, df = 1 (P = 0.03), I² = 78.8%								

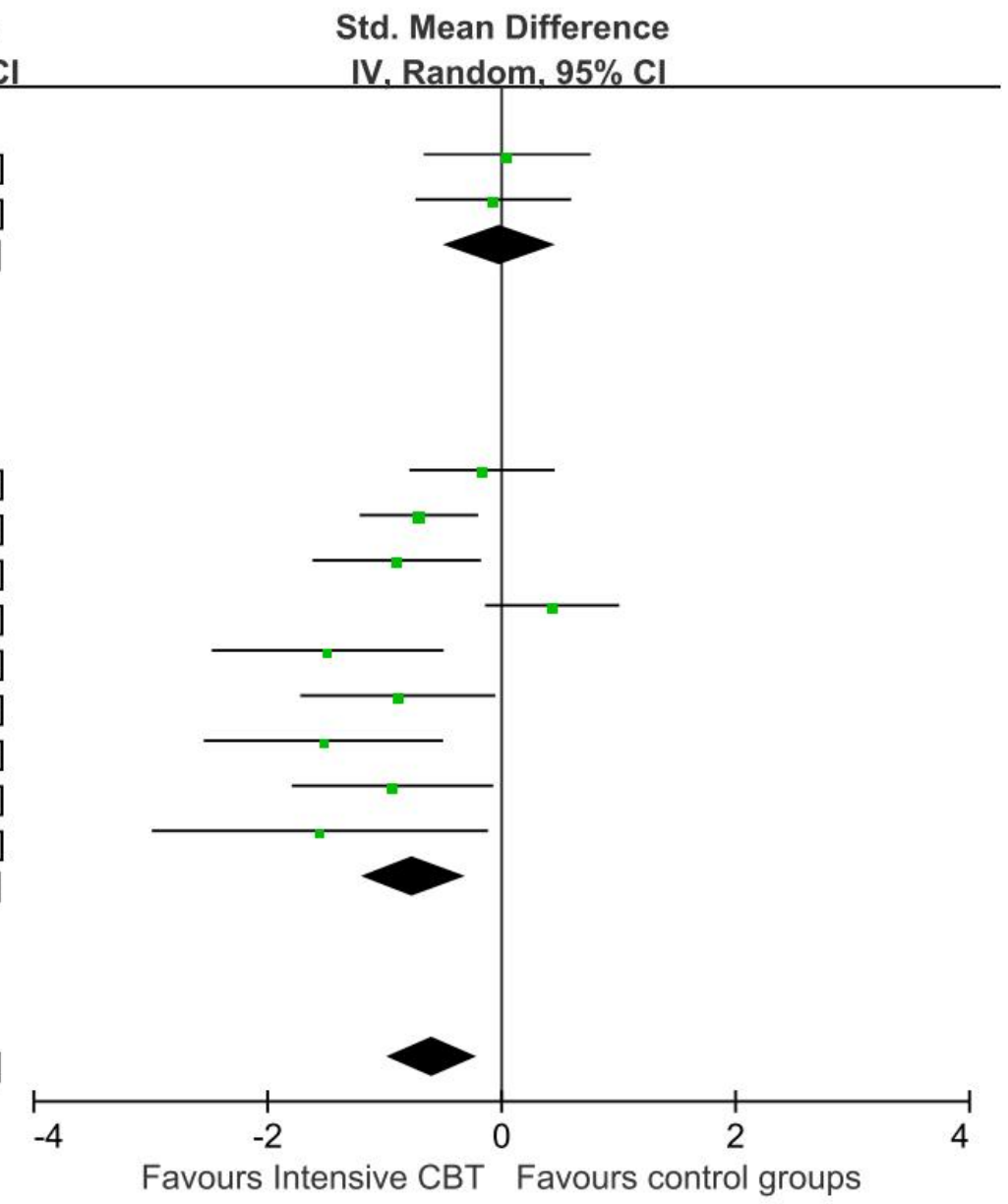

Supplementary Figure 9. Subgroup analysis of intensive CBT effects on OCD symptoms by age group (children vs. adults) at follow-up.

Study or Subgroup	Intensive CBT Mean	SD	Total	Control Mean	SD	Total	Weight	Std. Mean Difference IV, Random, 95% CI
Abramowitz, 2003	9.65	6.3	20	12.8	10.1	20	14.2%	-0.37 [-0.99, 0.26]
Barros, 2024	10.27	7.22	15	8.62	6.49	15	12.8%	0.23 [-0.48, 0.95]
Cottraux, 2001	2.1	6.2	32	5.6	5.8	30	16.1%	-0.58 [-1.08, -0.07]
Launes, 2019a	5.81	4.37	8	12.48	3.21	16	9.2%	-1.78 [-2.79, -0.77]
Launes 2019b	5.81	4.37	8	12.63	5.06	16	9.9%	-1.36 [-2.30, -0.41]
Lindsay, 1997	13.11	8.3	9	18.33	9.66	9	9.9%	-0.55 [-1.50, 0.39]
Storch, 2007	7.8	9	20	8.6	6.2	20	14.3%	-0.10 [-0.72, 0.52]
Vogel, 2004	10.6	9.2	16	9.7	8.3	18	13.5%	0.10 [-0.57, 0.77]
**Total (95% CI)**			**128**			**144**	**100.0%**	**-0.47 [-0.87, -0.06]**
Heterogeneity: Tau² = 0.20; Chi² = 17.63, df = 7 (P = 0.01); I² = 60%								
Test for overall effect: Z = 2.26 (P = 0.02)								

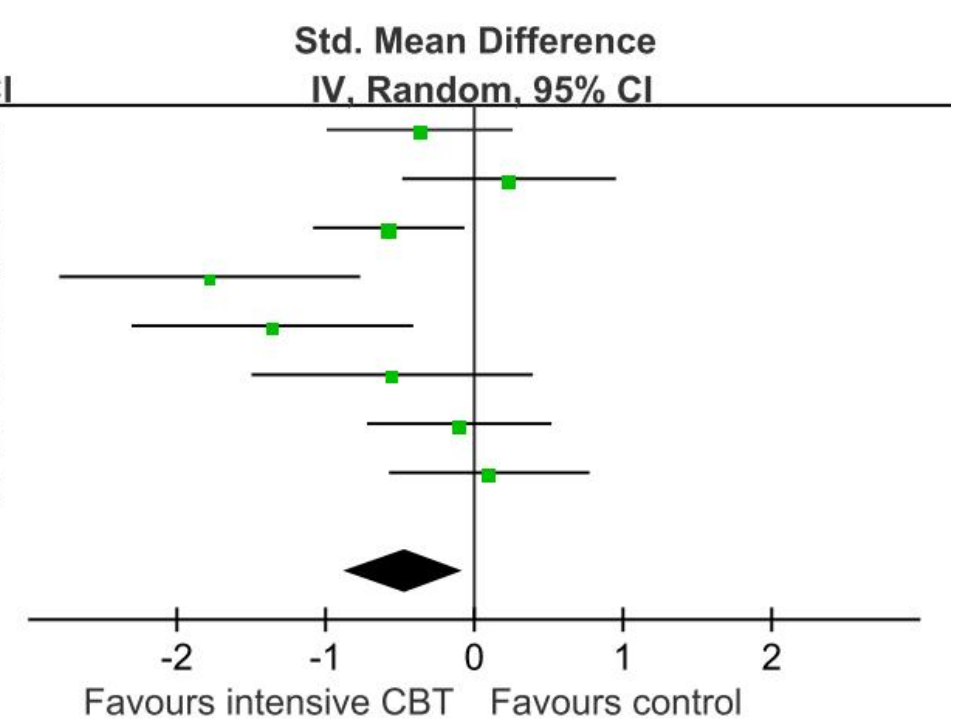

Supplementary Figure 10. Effect sizes for intensive CBT on depression symptoms vs. control groups at post-treatment.

Study or Subgroup	Intensive CBT Mean	SD	Total	Control Mean	SD	Total	Weight	Std. Mean Difference IV, Random, 95% CI
Barros, 2024	26.38	12.55	15	37.64	17.93	15	14.8%	-0.71 [-1.45, 0.03]
Launes, 2019a	6.81	3.89	8	12.24	4.81	16	12.9%	-1.16 [-2.08, -0.23]
Launes 2019b	6.81	3.89	8	12.44	5.1	16	12.9%	-1.14 [-2.06, -0.23]
Lewin, 2014	11.53	3.06	17	15.43	2.31	14	14.2%	-1.38 [-2.18, -0.58]
Lindsay, 1997	44.33	8.97	9	52.11	13.77	9	12.6%	-0.64 [-1.59, 0.32]
Storch, 2007	34.8	17.3	30	34.3	13.1	34	17.4%	0.03 [-0.46, 0.52]
Vogel, 2004	45.8	12.8	16	38.8	11.9	18	15.3%	0.55 [-0.13, 1.24]
**Total (95% CI)**			**103**			**122**	**100.0%**	**-0.59 [-1.14, -0.04]**
Heterogeneity: Tau² = 0.39; Chi² = 22.17, df = 6 (P = 0.001); I² = 73%								
Test for overall effect: Z = 2.09 (P = 0.04)								

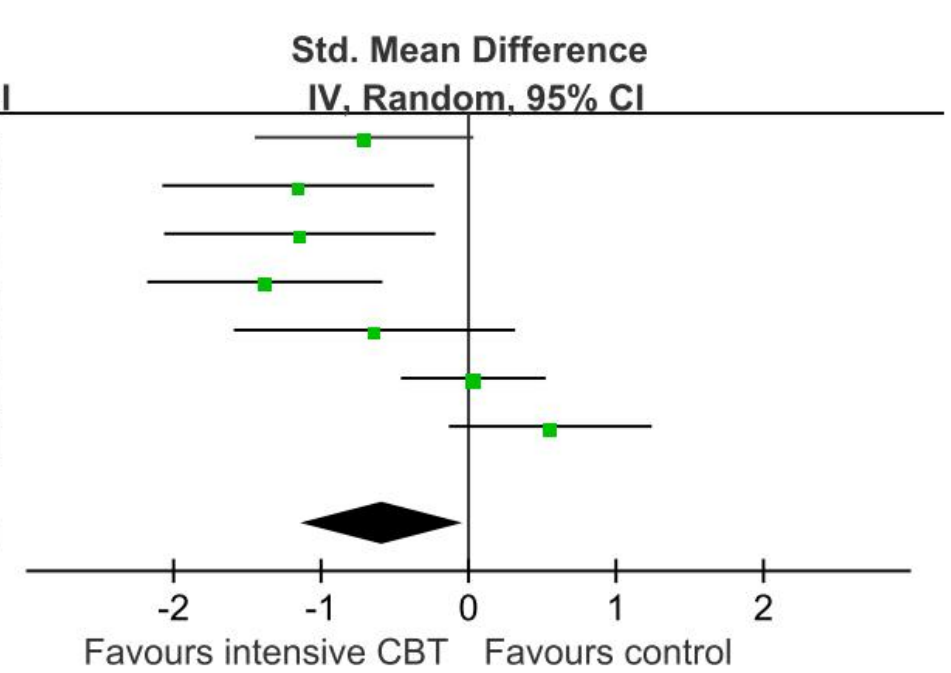

Supplementary Figure 11. Effect sizes for intensive CBT on anxiety symptoms vs. control groups at post-treatment.

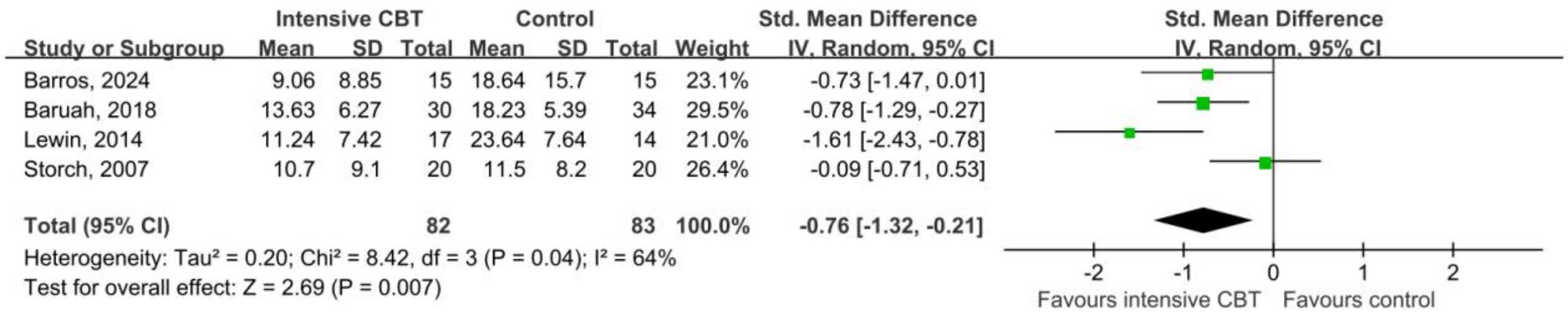

Supplementary Figure 12. Effect sizes for intensive CBT on family accommodation vs. control groups at post-treatment.

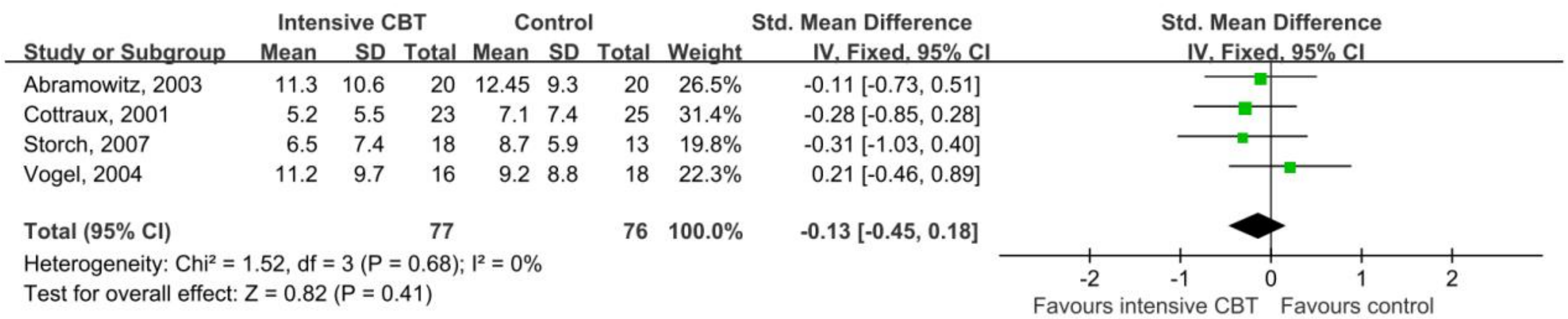

Supplementary Figure 13. Effect sizes for intensive CBT on depression symptoms vs. control groups at follow-up.

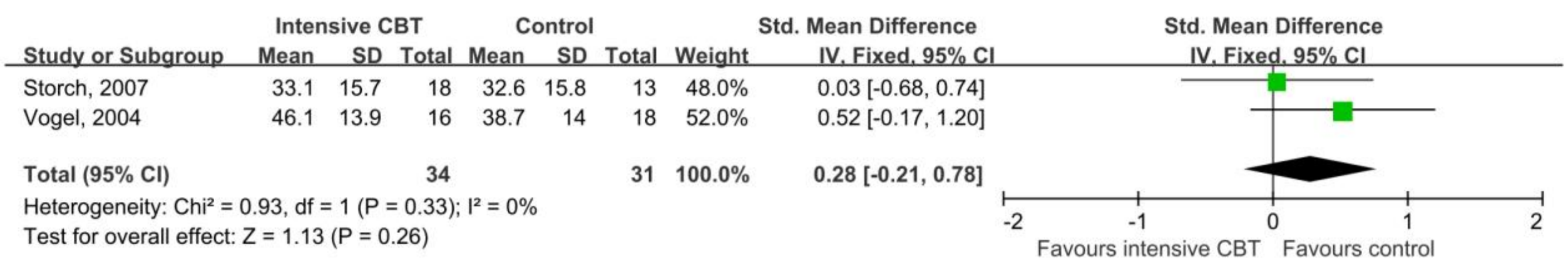

Supplementary Figure 14. Effect sizes for intensive CBT on anxiety symptoms compared vs. control groups at follow-up.

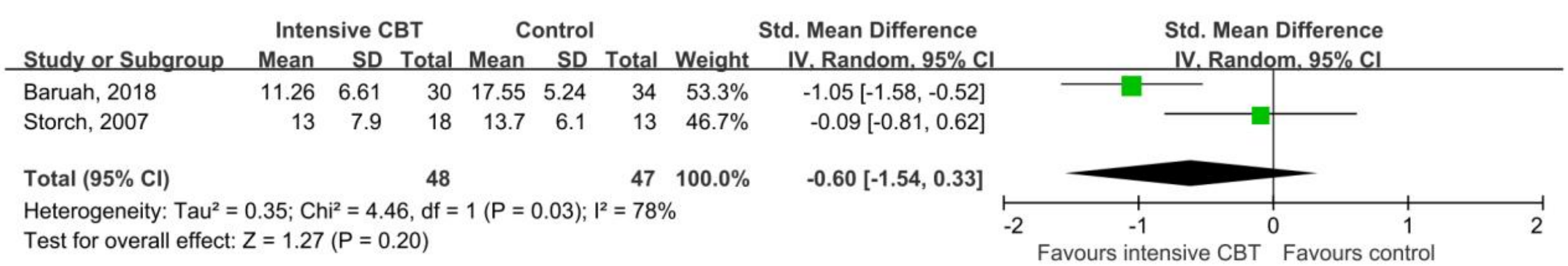

Supplementary Figure 15. Effect sizes for intensive CBT on family accommodation vs. control groups at follow-up.

Intensive CBT compared to [control treatment] for patients with OCD

Patient or population: patients with OCD
Setting: clinical
Intervention: intensive CBT
Comparison: [control treatment]

Outcomes	Anticipated absolute effects* (95% CI)		Relative effect (95% CI)	№ of participants (studies)	Certainty of the evidence (GRADE)	Comments
	Risk with [control treatment]	Risk with intensive CBT				
OCD (intensive CBT vs non-active condition)		**1.74 lower** (2.53 lower to 0.95 lower)	-	211 (6 RCTs)	⊕◯◯◯ Very low [b,c,f]	We have very little confidence in the effect estimate: the true effect is likely to be substantially different from the estimate of effect.
OCD (intensive CBT vs psychotherapy)		**0.17 lower** (0.47 lower to 0.14 higher)	-	177 (4 RCTs)	⊕◯◯◯ Very low [b,c,d]	We have very little confidence in the effect estimate: the true effect is likely to be substantially different from the estimate of effect.
OCD (intensive CBT vs pharmacotherapy)		**0.72 lower** (1.61 lower to 0.16 higher)	-	213 (2 RCTs)	⊕◯◯◯ Very low [b,c,d]	We have very little confidence in the effect estimate: the true effect is likely to be substantially different from the estimate of effect.
OCD symptoms	-	SMD **1 SD lower** (1.43 lower to 0.56 lower)	-	617 (12 RCTs)	⊕◯◯◯ Very low [a,f]	We have very little confidence in the effect estimate: the true effect is likely to be substantially different from the estimate of effect.
depression	-	SMD **0.47 SD lower** (0.87 lower to 0.06 lower)	-	272 (7 RCTs)	⊕◯◯◯ Very low [b,c,e]	We are very uncertain about the short-term effect of intensive CBT on depression.
anxiety	-	SMD **0.59 SD lower** (1.14 lower to 0.04 lower)	-	225 (6 RCTs)	⊕◯◯◯ Very low [b,c,e]	We are very uncertain about the short-term effect of intensive CBT on anxiety.
Family accommodation	-	SMD **0.76 SD lower** (1.32 lower to 0.21 lower)	-	165 (4 RCTs)	⊕◯◯◯ Very low [b,c,e]	We are very uncertain about the short-term effect of intensive CBT on FA.

***The risk in the intervention group** (and its 95% confidence interval) is based on the assumed risk in the comparison group and the **relative effect** of the intervention (and its 95% CI).

CI: confidence interval; **SMD:** standardized mean difference

Note: GRADE Working Group grades of evidence are as follow: High certainty = we are very confident that the true effect lies close to that of the estimate of the effect. Moderate certainty = we are moderately confident in the effect estimate: the true effect is likely to be close to the estimate of the effect, but there is a possibility that it is substantially different. Low certainty = our confidence in the effect estimate is limited: the true effect may be substantially different from the estimate of the effect. Very low certainty = we have very little confidence in the effect estimate: the true effect is likely to be substantially different from the estimate of effect.

[a] Publication bias was strongly suspected.
[b] Publication bias could not be reliably assessed due to the small number of studies.
[c] The total sample size was below the optimal information size (<400 participants for continuous outcomes).
[d] The confidence interval crossed the decision threshold.
[e] I^2 between 50% and 75% indicated substantial heterogeneity.
[f] $I^2 \geq 75\%$ indicated considerable heterogeneity.

Supplementary Figure 16. Summary of findings tables at post-treatment.

Intensive CBT compared to control treatment for patients with OCD

Patient or population: patients with OCD
Setting: clinical
Intervention: intensive CBT
Comparison: control treatment

Outcomes	Anticipated absolute effects* (95% CI)		Relative effect (95% CI)	№ of participants (studies)	Certainty of the evidence (GRADE)	Comments
	**Risk with control treatment**	**Risk with intensive CBT**				
OCD (intensive CBT vs non-active condition)		**0.97 lower** (1.35 lower to 0.59 lower)	-	138 (3 RCTs)	⊕⊕◯◯ Low [b,c]	Our confidence in the effect estimate is limited: the true effect may be substantially different from the estimate of the effect.
OCD (intensive CBT vs psychotherapy)		**0.08 higher** (0.24 lower to 0.4 higher)	-	154 (4 RCTs)	⊕◯◯◯ Very low [b,c,d]	We have very little confidence in the effect estimate: the true effect is likely to be substantially different from the estimate of effect.
OCD (intensive CBT vs pharmacotherapy)		**1.01 lower** (1.56 lower to 0.45 lower)	-	60 (2 RCTs)	⊕⊕◯◯ Low [b,c]	Our confidence in the effect estimate is limited: the true effect may be substantially different from the estimate of the effect.
OCD symptoms	-	SMD **0.59 SD lower** (0.98 lower to 0.21 lower)	-	352 (8 RCTs)	⊕◯◯◯ Very low [a,c,e]	We have very little confidence in the effect estimate: the true effect is likely to be substantially different from the estimate of effect.
depression	-	SMD **0.13 SD lower** (0.45 lower to 0.18 higher)	-	153 (4 RCTs)	⊕◯◯◯ Very low [b,c,d]	We are very uncertain about the long-term effect of intensive CBT on depression.
anxiety	-	SMD **0.28 SD higher** (0.21 lower to 0.78 higher)	-	65 (2 RCTs)	⊕◯◯◯ Very low [b,c,d]	We are very uncertain about the long-term effect of intensive CBT on anxiety.
FA	-	SMD **0.6 SD lower** (1.54 lower to 0.33 higher)	-	95 (2 RCTs)	⊕◯◯◯ Very low [b,c,d,f]	We are very uncertain about the long-term effect of intensive CBT on FA.

***The risk in the intervention group** (and its 95% confidence interval) is based on the assumed risk in the comparison group and the **relative effect** of the intervention (and its 95% CI).

CI: confidence interval; **SMD:** standardized mean difference

Note: GRADE Working Group grades of evidence are as follow: High certainty = we are very confident that the true effect lies close to that of the estimate of the effect. Moderate certainty = we are moderately confident in the effect estimate: the true effect is likely to be close to the estimate of the effect, but there is a possibility that it is substantially different. Low certainty = our confidence in the effect estimate is limited: the true effect may be substantially different from the estimate of the effect. Very low certainty = we have very little confidence in the effect estimate: the true effect is likely to be substantially different from the estimate of effect.

[a] Publication bias was strongly suspected.
[b] Publication bias could not be reliably assessed due to the small number of studies.
[c] The total sample size was below the optimal information size (<400 participants for continuous outcomes).
[d] The confidence interval crossed the decision threshold.
[e] I^2 between 50% and 75% indicated substantial heterogeneity.
[f] $I^2 \geq 75\%$ indicated considerable heterogeneity.

Supplementary Figure 17. Summary of findings tables at follow-up.
